# Identification and Preliminary Structure-Activity Relationship Studies of 1,5-Dihydrobenzo[*e*][1,4]oxazepin-2(3*H*)-ones That Induce Differentiation of Acute Myeloid Leukemia Cells In Vitro

**DOI:** 10.3390/molecules26216648

**Published:** 2021-11-02

**Authors:** Laia Josa-Culleré, Thomas J. Cogswell, Irene Georgiou, Morgan Jay-Smith, Thomas R. Jackson, Carole J. R. Bataille, Stephen G. Davies, Paresh Vyas, Thomas A. Milne, Graham M. Wynne, Angela J. Russell

**Affiliations:** 1 Department of Chemistry, Chemistry Research Laboratory, University of Oxford, Mansfield Road, Oxford OX1 3TA, UK; tom.cogs1@gmail.com (T.J.C.); igeorgiou001@dundee.ac.uk (I.G.); morgan.jaysmith@outlook.com (M.J.-S.); carole.bataille@pharm.ox.ac.uk (C.J.R.B.); steve.davies@chem.ox.ac.uk (S.G.D.); graham.wynne@yahoo.co.uk (G.M.W.); 2 MRC Molecular Haematology Unit, MRC Weatherall Institute of Molecular Medicine, NIHR Oxford Biomedical Research Centre Haematology Theme, Radcliffe Department of Medicine, University of Oxford, Oxford OX3 9DS, UK; thomas.jackson@paediatrics.ox.ac.uk (T.R.J.); paresh.vyas@imm.ox.ac.uk (P.V.); thomas.milne@imm.ox.ac.uk (T.A.M.); 3 Department of Pharmacology, University of Oxford, Mansfield Road, Oxford OX1 3QT, UK

**Keywords:** acute myeloid leukemia, differentiation, phenotypic screen, CD11b, benzooxazepinones

## Abstract

Acute myeloid leukemia (AML) is the most aggressive type of blood cancer, and there is a continued need for new treatments that are well tolerated and improve long-term survival rates in patients. Induction of differentiation has emerged as a promising alternative to conventional cytotoxic chemotherapy, but known agents lack efficacy in genetically distinct patient populations. Previously, we established a phenotypic screen to identify small molecules that could stimulate differentiation in a range of AML cell lines. Utilising this strategy, a 1,5-dihydrobenzo[*e*][1,4]oxazepin-2(3*H*)-one hit compound was identified. Herein, we report the hit validation in vitro, structure-activity relationship (SAR) studies and the pharmacokinetic profiles for selected compounds.

## 1. Introduction

Acute myeloid leukemia (AML) is a type of blood cancer characterised by a block in differentiation of cells of the myeloid lineage. Their abnormal growth and differentiation lead to an accumulation of immature myeloid precursors in the bone marrow and peripheral blood, which disrupts the formation of terminally differentiated blood cells. AML is a highly heterogeneous disease, which is often divided into genetic subtypes [1,2].

The current standard of care (SOC) consists of induction therapy using cytarabine and an anthracycline, followed by consolidation chemotherapy or allogeneic stem cell transplant [3]. The majority of AML cases are elderly (>65 years of age) and do not tolerate this intensive chemotherapy well. The SOC regimen achieves better responses in younger patients (<60 years of age), with 50–80% complete remission; however, 60–70% of these patients will relapse [3,4,5,6]. Thus, there is a clear need for new treatments that are better tolerated and that provide high long-term survival.

Differentiation therapy is an alternative approach to standard cytotoxic therapies and has recently gained much attention [7]. Such a therapy aims to relieve the differentiation block of AML cells, pushing them towards normal myeloid maturation. Early results with differentiation therapies suggest that in some circumstances they can be both less toxic and more effective than simply inducing cytotoxicity [7]. The first successful example of this approach was the combination of all-*trans* retinoic acid (ATRA) [8] and arsenic trioxide (ATO) [9] to treat acute promyelocytic leukemia (APL). Previously, this subtype of AML had a poor prognosis [10]; however, induction of differentiation with ATRA and ATO has greatly improved the outlook for patients, culminating in an 85% 5-year survival rate [11]. Although these are still early days, the hope is that these approaches could be expanded to a wider range of different AML patients.

Since the discovery of ATRA for the treatment of APL, various differentiating agents have been described, such as IDH1/2 [12,13] and LSD1 inhibitors [14]. Whilst these represent significant steps forward in terms of therapeutic options for patients, the limitation of these agents is that they target specific genetic lesions and are effective only in specific patient subtypes. Thus, we and others [15,16] have been interested in identifying compounds using alternative mechanisms and which are able to induce differentiation of AML cells, regardless of their subtype or mutation status. The hope is that such compounds will be effective in wider patient populations and will avoid some of the limitations of therapies designed for specific targets [17].

Within our effort to identify novel differentiating agents for AML, we developed a phenotypic screen with different AML cell lines representing different disease subtypes and measured the myeloid marker CD11b as a primary readout. We identified several compound classes, some of which showed in vivo efficacy [15,18]. Herein, we report the identification and structure-activity relationship (SAR) studies of one of these compound classes, which possesses a 1,5-dihydrobenzo[*e*][1,4]oxazepin-2(3*H*)-one core structure.

## 2. Results and Discussion

### 2.1. Identification of OXS003976 as a Confirmed Hit from a Phenotypic Screen

To identify small molecules able to differentiate AML cells with different mutation characteristics, we performed a phenotypic screen using 4 genetically distinct cell lines [2]: HL-60, THP-1, OCI-AML3 and KG-1. The expression of CD11b was selected as the primary readout, as CD11b is upregulated upon myeloid differentiation [19,20].

Cells were treated with 1000 structurally diverse small molecules selected from a commercial library [18]. A single point testing concentration of 10 µM was used. After 4 days, cells were stained with a CD11b antibody and analysed using flow cytometry. Compounds upregulating CD11b expression greater than 10% in at least three of the cell lines were considered as potential hits. Hit confirmation was undertaken as follows: first, the hit was re-tested at 10 µM both with the CD11b antibody and an isotype control to account for nonspecific binding. Secondly, the effect of the hit on cell proliferation and viability was evaluated through staining with DAPI to evaluate the number of dead cells, and with acridine orange to detect total cell numbers. Thirdly, the induction of differentiation caused by the compounds was confirmed through morphology assessment with Giemsa staining. Finally, the molecule was resynthesised and fully characterised to confirm structural authenticity. This fresh sample was then retested in the CD11b assay to generate a concentration-response curve. The study of proliferation and morphology was performed on a panel of six cell lines: HL-60, THP-1, OCI-AML3, KG-1, Kasumi-1 and ME-1.

**Figure 1:**
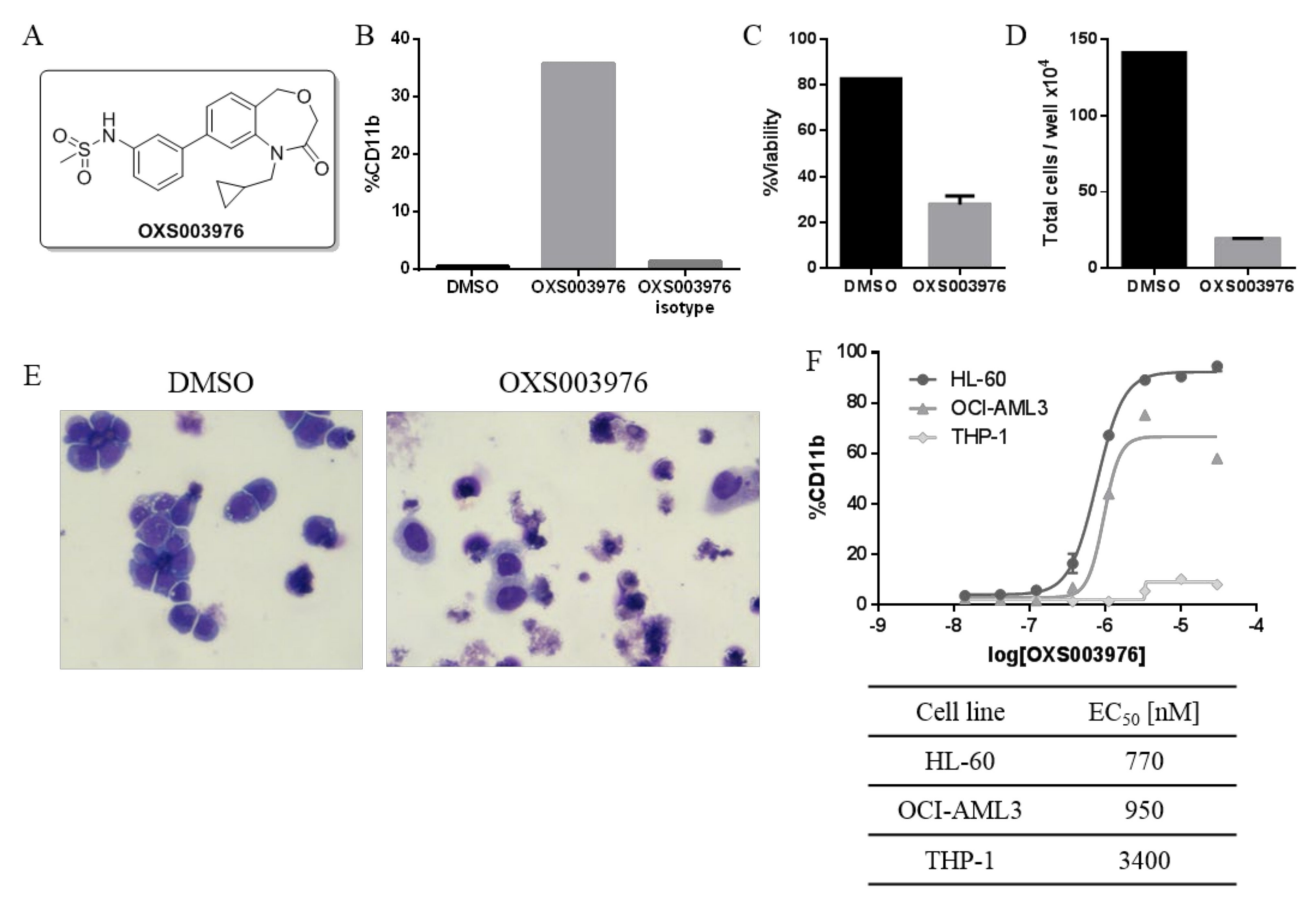
Profile of the confirmed hit OXS003976: (**A**) chemical structure of OXS003976; (**B**) OXS300976 (10 µM) upregulates CD11b expression but not of the isotype antibody after a 4-day treatment of HL-60 cells; (**C**) OXS003976 (10 µM) decreases viability and (**D**) total number of HL-60 cells after a 4-day treatment; (**E**) OXS003976 (10 µM) induces changes in the morphology of HL-60 cells after a 4-day treatment; (**F**) a resynthesised sample OXS003976 increases CD11b expression of HL-60, OCI-AML3 and THP-1 cells in a dose-dependent manner after a 4-day treatment.

One of the chemical entities identified and further confirmed as a hit from the screen was 1,5-dihydrobenzo[*e*][1,4]oxazepin-2(3*H*)-one OXS003976 **5** (Figure 1A). OXS003976 upregulated CD11b expression, but not the isotype control (Figure 1B), and decreased cell proliferation and viability (Figure 1C,D and Figure S1). Moreover, it induced morphological changes consistent with differentiation in the six cell lines (Figure 1E and Figures S2–S6), such as increased size, lighter cytoplasm and increased cytoplasm-to-nuclei ratio.

**Scheme 1:**
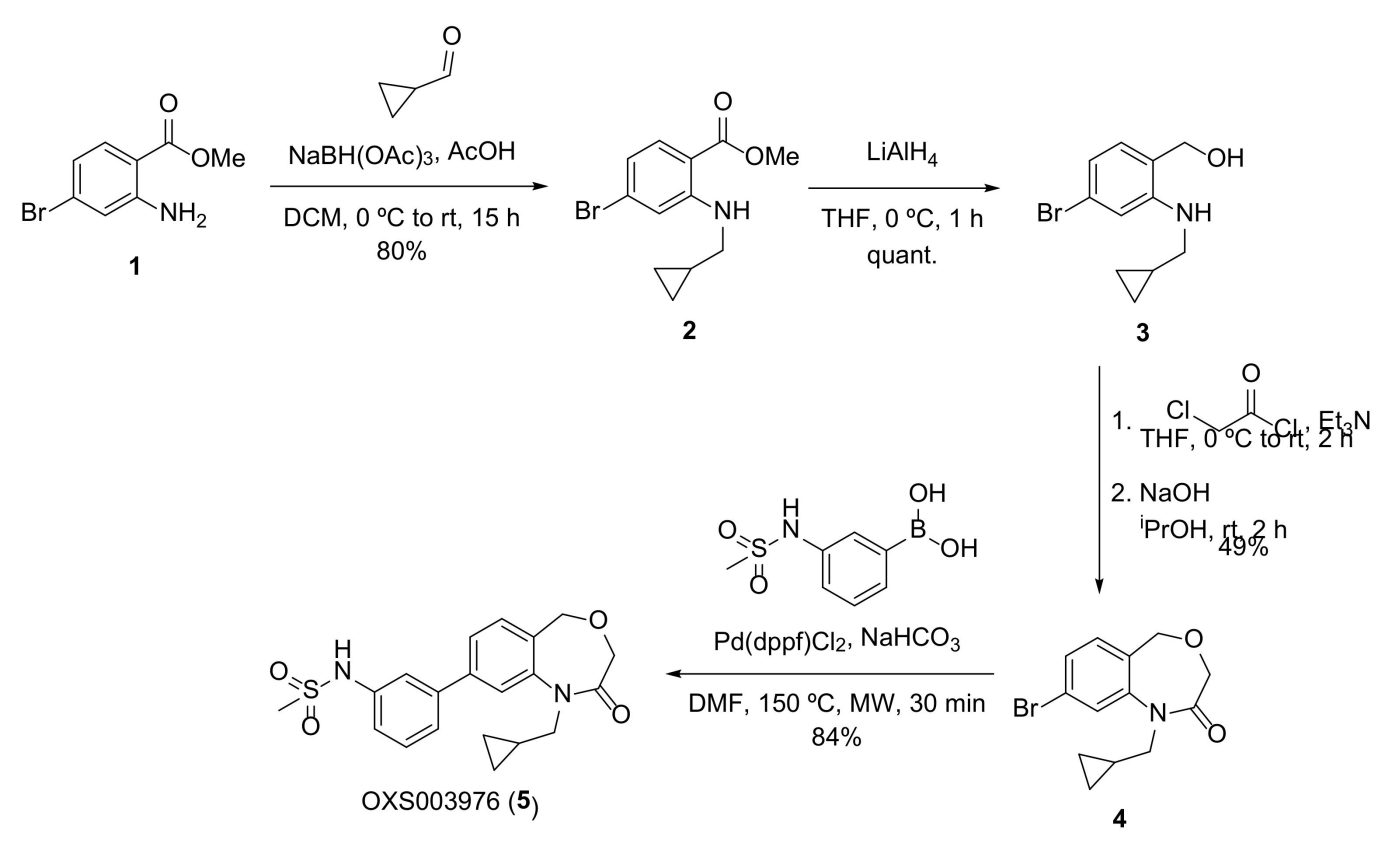
Synthesis of the hit OXS003976.

The resynthesis route used was analogous to one reported for similar compounds [21] substituted at the 7- instead of the 8-position of the dihydrobenzoxazepinone core (Scheme 1). Starting from commercially available methyl 2-amino-4-bromobenzoate **1**, the cyclopropylmethyl group was first introduced via reductive amination to provide **2**. Reduction of the methyl ester afforded alcohol **3**, which was cyclised through a 2-step process using chloroacetyl chloride followed by sodium hydroxide. This cyclisation can also be performed with bromoacetyl bromide followed by potassium *tert*-butoxide in similar yields. The target compound was obtained after a final Suzuki reaction at the 8-position.

Upon completion of the resynthesis, OXS003976 was found to upregulate CD11b expression in HL-60, OCI-AML3 and THP-1 in a concentration-dependent manner (Figure 1F, EC_50_ of 770, 950 and 3400 nM respectively), confirming its ability to remove the differentiation block in these genetically diverse AML cell lines.

### 2.2. Structure-Activity Relationship Studies

With the confirmed hit OXS003976 in hand, an initial assessment of its physicochemical properties revealed that it had high aqueous solubility (>200 µM) but relatively low metabolic stability in the mouse S9 fraction (extraction ratio (ER) [22] = 0.54).

Before this compound series could be considered for progression into in vivo evaluation, the priorities were to significantly improve both its moderate potency and metabolic stability. Thus, structure-activity relationship studies were initiated, where the potency of the new analogues was evaluated through concentration-response testing using the CD11b assay in HL-60 cells. Lipophilic efficiency (LipE), which also accounts for lipophilicity, was also used to compare the quality of the analogues. Even though multiple parameters can influence activity in a cell-based assay, LipE is a useful guide to compare analogues [23].

To improve the intrinsic potency of the hit, we first focused on modification of the *N*-1 substituent of the cyclic amide (Table 1). We also hypothesised that the *N*-cyclopropylmethyl moiety of OXS003976 could be a potential metabolic liability, thus we expected some of these compounds to have improved metabolic stability. In this first round of SAR it was found that biological activity was dependent on the steric bulk at this position. Both the nonsubstituted **6**, and **7**, substituted with a small methyl group, were completely inactive. Increasing size from a methyl (**7**) to an ethyl substituent (**8**) rescued some level of activity. Different branched (isopropyl OXS007002 **9**, isopropylmethyl **10**) and cyclic groups (cyclopentyl **12**) had similar levels of activity. From these set of analogues, *N*-isopropyl OXS007002 proved to be the most active (EC_50_ = 620 nM) and, encouragingly, also had improved metabolic stability relative to the starting compound (ER = 0.33), in addition to having the highest LipE (4.5).

In an attempt to further increase metabolic stability, *N*-trifluoroethyl **11** was also prepared. Encouragingly, a significant decrease in metabolic extraction ratio was observed (ER = 0.05); however, this was at the expense of activity (EC_50_ = 1.9 µM, LipE = 3.9). The slightly more lipophilic *N*-phenyl containing analogue **14** was found to be the most active in this series (EC_50_ = 220 nM), although with a lower LipE than isopropyl OXS007002.

**Scheme 2:**
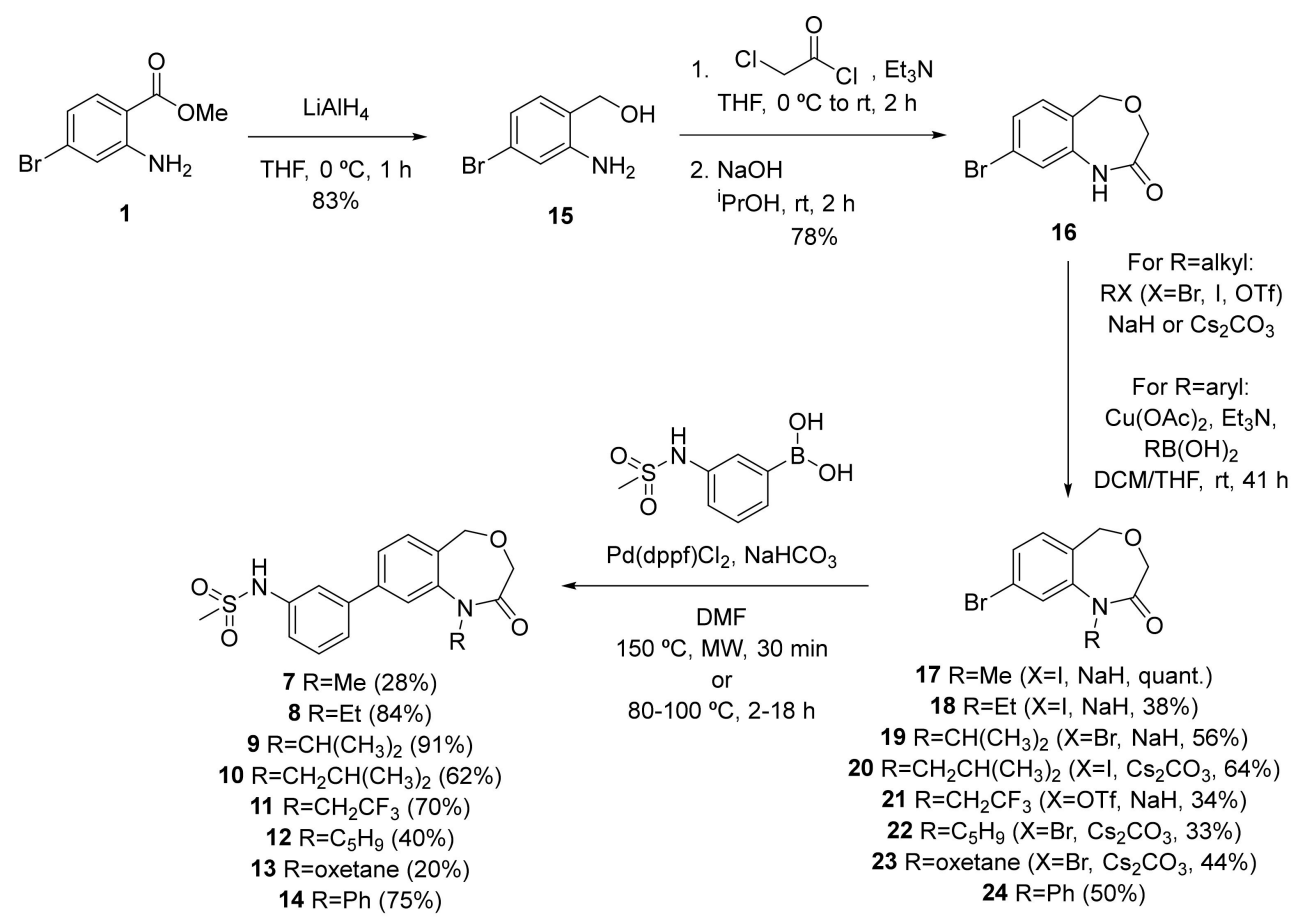
Synthesis of analogues with different *N*-1 substituents.

To facilitate the synthesis of these analogues, the route used for OXS003976 was modified to enable introduction of the *N*-substituents at a later stage (Scheme 2). Specifically, the dihydrobenzoxazepinone core was first cyclised to **16**, which was further derivatised via nucleophilic substitution. The exception was *N*-phenyl analogue **14**, in which the aromatic group was introduced through Chan-Lam coupling. Better yields for the final Suzuki reaction were obtained when performed after *N*-substitution.

With the aim of further improving the potency, we investigated modifications of the aromatic group at the 8-position (Table 2). The *N*-substituent that was used for these analogues was the isopropyl (e.g., compound **9**), which had the best balance of intrinsic potency vs. stability and the highest LipE.

Varying the location of the sulfonamide to *ortho* **25** and *para* **26** confirmed that substitution in the *meta* position (OXS007002) was optimal. Examining this position further, changing the *meta* substituent to the sulfonamide bioisostere sulfoximine [24] **27** removed the activity, while carbamate **28** was equally active. However, the carbamate did not seem to offer an advantage over the sulfonamide as it had similar levels of metabolic stability but lower solubility. Alternatively, electron donating (**29**, **30**, **31**) or electron withdrawing (**32**) groups also led to a decrease or loss of activity.

We also explored whether extending the sulfonamide alkyl group could improve the intrinsic potency of these compounds. However, the activity of *n*-propyl **33** decreased potency by 10-fold compared to OXS007002. With the same purpose, an additional substituent in the *para* position was trialed. In this case, fluoro **35**, methyl **36** and methoxy **37** all retained some activity, and in particular methyl **36**, albeit with a loss in metabolic stability. Additional modifications incorporating an ether linker (**40**) or replacing the phenyl with a pyridine (**39**) abolished activity.

Thus, while substitution at the *N*-position proved to be somewhat flexible, with various analogues showing similar levels of biological activity, the opposite was found at the 8-position, where small modifications often led to a significant or complete loss of activity.

Having found that the *meta*-sulfonamide was the optimal substituent amongst those trialed, we turned our attention to the core of the molecule (Table 3). The 6,6-fused ring analogue **41** retained caused a 15-fold loss in activity when compared to OXS003976, confirming that the original oxazepanone was preferred. We also found that pyridine analogue **42** retained weak activity, whilst greatly improving metabolic stability, and the LipE of 4.9 was the highest amongst the compounds reported herein.

**Scheme 3:**
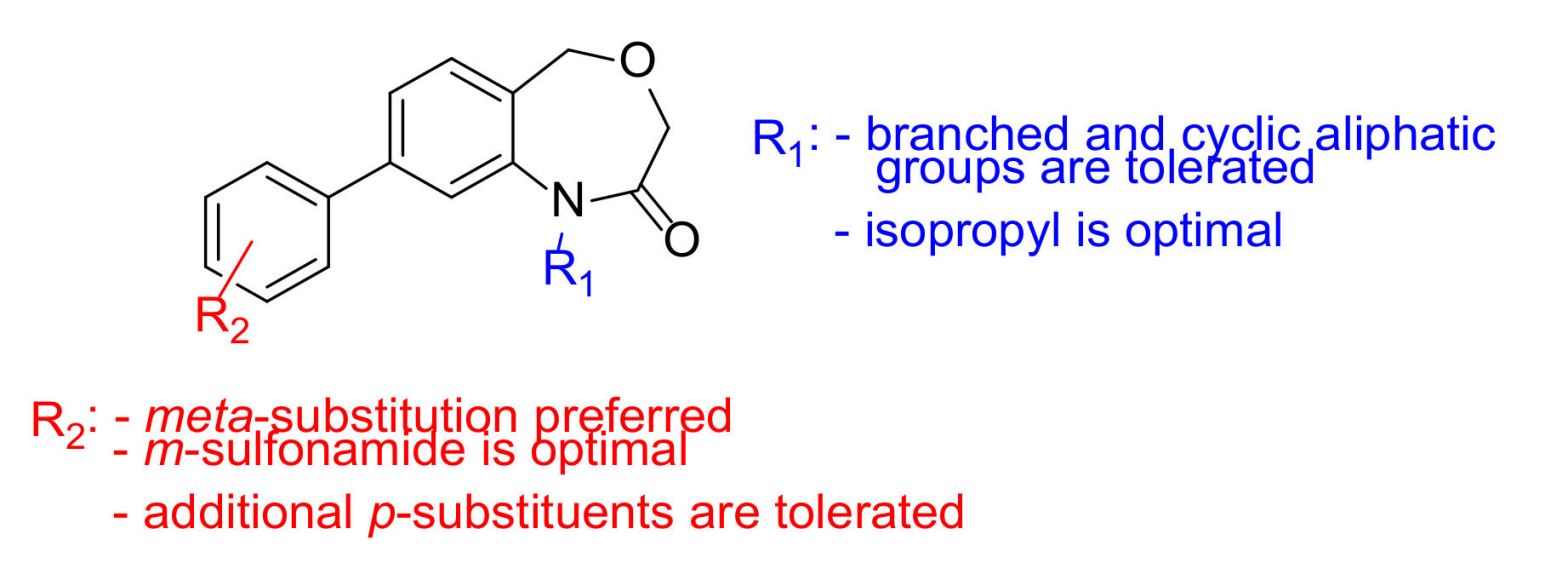
Structure-activity summary for the core 1,5-dihydrobenzo[*e*][1,4]oxazepin-2(3*H*)-one.

Overall, SAR studies of a series of 1,5-dihydrobenzo[*e*][1,4]oxazepin-2(3*H*)-one analogues showed that the optimal substituent at *N*-1 is an isopropyl, which gives the highest activity and LipE from the compounds reported (Scheme 3). Other aliphatic groups are also tolerated. With regards to the 8-position, substitution of the aryl group at the *meta*-position is necessary for activity, and the sulphonamide and carbamate groups show the highest potencies; the former has higher solubility. An additional small *p*-substituent (F, Me, OMe) can also be incorporated with no small effect on activity.

### 2.3. Assessment of Pharmacokinetic Profile

**Figure 2:**
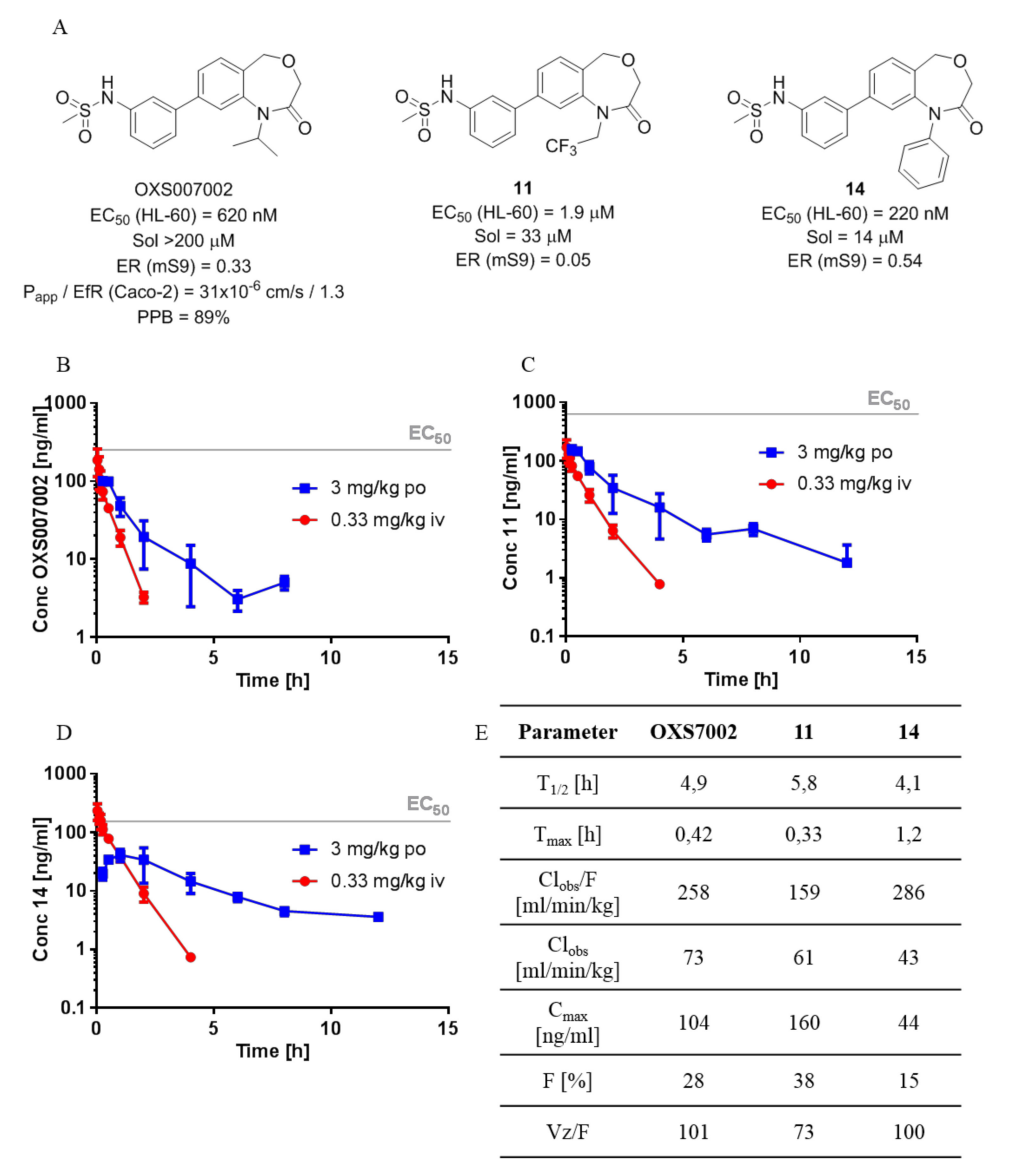
(**A**) Compounds selected for PK studies. Pharmacokinetic profile of (**B**) OXS007002, (**C**) **11** and (**D**) **14** after a single dose of 3 mg/kg (po) and 0.33 mg/kg (ip). (**E**) Pharmacokinetic parameters.

In order to undertake a preliminary assessment of the pharmacokinetic (PK) profile of this compound class, three compounds were deemed to have sufficient intrinsic potency, solubility and in vitro metabolic stability properties to progress into a single dose in vivo PK study. Isopropyl bearing example OXS007002 had the second highest LipE and was also found to have high Caco-2 cell permeability (P_app_ of 31 × 10^−6^ cm/s), a low efflux ratio (EfR of 1.3), and acceptable plasma protein binding (PPB, 11% unbound). Trifluoroethyl **11**, which had the highest metabolic stability, and phenyl substituted **14**, which was the most active, were also progressed (Figure 2A). Male CD-1 mice were administered with a single dose of each compound, both intravenous (iv) at 0.33 mg/kg and orally (po) at 3 mg/kg, and the compound concentration in blood was measured over 24 h (Figure 2). Compound **14** was found to have the lowest clearance, correlating well with its low S9 extraction ratio. Nonetheless, the clearance of the three compounds was high to moderate, and at this dose the in vitro EC_50_ was not reached. From these results, it was clear that further optimisation of the compounds was needed to further improve potency, physicochemical and metabolic characteristics, before progressing into any other in vivo studies.

### 2.4. Target Identification Studies

To gain some insights into the possible pharmacological target(s) driving the differentiation effect of this compound class, OXS007002 was tested against previously reported targets of differentiation, such as DHODH and FLT3, and a panel of kinases. The compound was found to be inactive at testing concentrations from 1 to 10 μM suggesting an alternative mechanism of action. We have recently found that microtubule disruptors can induce differentiation of AML cells [18]. Thus, OXS007002 was tested for its effect on tubulin polymerisation in a cell-free assay [25,26] and found to inhibit it with an IC_50_ of 3.6 μM (Figure S7A). Conversely, the close structural analogue *ortho*-**25**, which was inactive in the CD11b expression assay, did not have an effect on polymerisation of tubulin at testing concentrations of up to 100 μM (Figure S7B). These results suggest that this compound class could be inducing differentiation of AML cells through tubulin disruption; however, more studies would be needed to confirm that tubulin is driving the effect, and whether there are other pathways involved.

## 3. Conclusions

In this work, we identified a series of 1,5-dihydrobenzo[*e*][1,4]oxazepin-2(3*H*)-ones that are able to differentiate AML cell lines representing different patient subtypes. The initial hit, which was identified through a phenotypic screen, was confirmed to have differentiation effects through expression of CD11b, a block in proliferation and changes in cell morphology. Structure-activity relationship studies identified additional analogues with increased potency and LipE. Subsequent PK studies showed that after oral dosing the compounds tested did not demonstrate sufficiently high plasma levels to progress to efficacy studies. Nonetheless, we describe a novel class of differentiation agents alongside SAR analysis, which may allow further optimisation to improve potency, physicochemical properties and PK profiles.

## 4. Materials and Methods

### 4.1. Cell Culture

AML cell lines HL-60, THP-1, OCI-AML3, Kasumi-1, KG-1 and ME-1 were purchased from the American Type Culture Collection (ATCC). The cells were maintained in RPMI supplemented with 10% fetal bovine serum and 1% L-glutamine.

### 4.2. Flow Cytometry

PE mouse-anti-human-CD11b/Mac-1 (ICRF44, Cat 555388) and PE-mouse-IgG1-κ-isotype control (MOPC-21, Cat 555749) were purchased from BD Bioscience. Cells were suspended in 100 μL growth media (RPMI + 10% FBS + 1% *L*-glutamine) at a density of 2 × 10^4^ cells/well of a V-bottom 96 well plate (Corning, Cat 3894) and grown for 96 h in the presence of compound at the required concentration. After 96 h, the plate was centrifuged, media was removed, and cells were resuspended in 50 µL of blocking buffer (IMDM, no phenol red + 10% FBS) containing 2.5 μL of either CD11b or isotype control antibody and incubated on ice in the dark for 20 min. Cells were washed twice in 200 μL of staining buffer (IMDM, no phenol red + 1% FBS) before being resuspended in 200 μL of staining buffer containing 1 μg/mL of DAPI to help identify dead cells. Flow cytometry data were collected on an Attune NxT and analysed using the Attune NxT software (Life Technologies).

### 4.3. Cell Counts and Viability Assessment

Solution 13 containing acridin orange and DAPI was purchased from ChemoMetec (Cat 910-3013). After the appropriate cell treatment, one volume of solution 13 was added into 19 volumes of the premixed cell suspension and analysed using the NucleoCounter^®^ NC-300^TM^ (ChemoMetec).

### 4.4. Morphology Assessment with Wright Staining

Cells were prepared in staining buffer (IMDM, no phenol red + 2% FBS) at a concentration of approximately 1 × 10^5^ cells/mL. Cytospins were made (1000 rpm, 10 min), and the cells were allowed to air dry. Cells were stained with Modified Wright stain using a Hematek^®^. Stained cells were allowed to air dry and coverslips were affixed with DPX mount prior to microscopy.

### 4.5. ADME Properties

Determination of semi-thermodynamic solubility, metabolic stability in S9 fraction, Caco-2 permeability and PPB were performed by Cyprotex Ltd. (www.cyprotex.com, accessed on 22 October 2021) using their standard assays and as described on their website.

### 4.6. PK Studies

Male CD-1 mice were used for the PK studies, with three for each treatment. The three compounds were dosed either PO at 3 mg/kg in 5:94.9:0.1 *v*/*v*/*v* DMSO/PBS/Tween 20, or IV at 1 mg/kg in 5:95 *v*/*v* DMSO/HPCD (20% *w*/*v*). Blood concentration was measured by LC-MS/MS over 24 h.

### 4.7. Microtubule Polymerisation Assay

The microtubule polymerisation assay was performed using porcine neuronal tubulin (Cytoskeleton, Inc, Denver, CO, USA, BK006P) as an adaptation of the original method of Shelanski et al. [25] and Lee et al. [26] at Cytoskeleton, Inc.

### 4.8. Chemical Synthesis General Information

All reactions involving moisture sensitive reagents were carried out under a nitrogen or an argon atmosphere. Anhydrous solvents were dried by passing over an activated alumina column under an inert atmosphere using a solvent purification system. All other solvents and reagents were used as supplied (analytical or HPLC grade) without prior purification. Flash column chromatography was performed on Kieselgel 60 silica gel (230–400 mesh particle size) on a glass column or on a Biotage SP4 automated flash column chromatography platform. NMR spectra were recorded on a Bruker Advance spectrometers at 400 or 500 MHz in the stated deuterated solvent at room temperature. The field was locked by external referencing to the relevant deuteron resonance. Chemical shifts (δ) are reported in parts per million (ppm) and coupling constants (*J*) are quoted in Hz. Data are reported as follows: chemical shift, multiplicity (s = singlet, d = doublet, t = triplet, q = quartet, sext = sextuplet, hept = heptet, and m = multiplet), coupling constant and integration. Low-resolution mass spectra (*m/z*) were recorded on an Agilent 1260 Infinity II with Diode Array and Single Quadrupole Detectors in solutions of MeOH. Each selected peak is reported in Daltons and its intensity given as percentage of the base peak. High resolution mass spectra (HRMS) were run on a Bruker microTOF (ESI and APCI) or on a Waters GCT (EI). Purity of the biologically tested compounds was >95% based on NMR and HPLC.

### 4.9. General Procedure A: Reductive Amination

Sodium acetoxyborohydride (2 equiv.) was added portion-wise to a solution of methyl 2-amino-4-bromobenzoate (1 equiv.), the required aldehyde (1.5 equiv.) and acetic acid (0.5 equiv.) in anhydrous DCM (*c* 0.2 M) at 0 °C. The mixture was allowed to warm to room temperature and stirred at this temperature for 15 h. The reaction mixture was diluted with DCM, quenched with saturated aqueous NaHCO_3_, extracted with 3×DCM, dried over anhydrous Na_2_SO_4_ and concentrated in vacuo to produce the desired amine, which was taken to the following step without further purification.

### 4.10. General Procedure B: Ester Reduction

Lithium aluminium hydride (1 M in THF, 3.5 equiv.) was added dropwise to a solution of the required ester (1.0 equiv.) in THF (*c* 0.15 M) at 0 °C. After stirring at room temperature for 1 h, the reaction mixture was quenched with saturated aqueous NH_4_Cl at 0 °C, extracted with 3×EtOAc, dried over anhydrous Na_2_SO_4_ and concentrated *in vacuo* to produce the desired alcohol, which was taken to the following step without further purification.

### 4.11. General Procedure C.1: Cyclisation with Chloroacetyl Chloride

Chloroacetyl chloride (4.4 equiv.) was added to a solution of the required alcohol (1 equiv.) and triethylamine (2 equiv.) in THF (*c* 0.25 M) at 0 °C. The mixture was stirred at room temperature for 2 h, then passed through Celite, washed with EtOAc and concentrated under reduced pressure. The crude residue was then dissolved in IPA (*c* 0.25 M), and sodium hydroxide (2.5 equiv.) was added. The mixture was stirred at room temperature for 2 h, then diluted with DCM, washed with brine, dried over anhydrous Na_2_SO_4_ and concentrated *in vacuo*. The crude product was purified by flash column chromatography to produce the desired 1,5-dihydrobenzo[*e*][1,4]oxazepin-2(3*H*)-one.

### 4.12. General Procedure C.2: Cyclisation with Bromoacetyl Bromide

Bromoacetyl bromide (1.1 equiv.) was added to a solution of the required alcohol (1 equiv.) in DCM (*c* 0.1 M) at 0 °C. After stirring for 10 min at 0 °C, 2 M aqueous Na_2_CO_3_ (10 equiv.) was added over a period of 10 min. The cooling bath was removed and stirring was continued for 18 h. The reaction mixture was concentrated under vacuum and the aqueous residue was extracted with EtOAc. The combined organic layers were washed with brine, dried and concentrated under vacuum. The resulting crude product was dissolved in 2-propanol (*c* 0.1 M), and KO*^t^*Bu (1.5 equiv.) was added portion-wise at 0 °C. The reaction mixture was stirred overnight and subsequently quenched with water. The aqueous layer was extracted with EtOAc and the combined organic extracts were dried, filtered and concentrated under vacuum to produce the desired product

### 4.13. General Procedure D: Suzuki Reaction

A solution of the required bromide (1 equiv.) in DMF/water (1:1, *c* 0.1 M) under argon was added sodium hydrogen carbonate (3 equiv.), the desired boronic acid or pinacol ester (1.3 equiv.) and Pd(dppf)Cl_2_ (0.05 equiv.), and the mixture was degassed with argon. The mixture was heated at 80–100 °C overnight or in the microwave at 150 °C for 30 min. Upon completion, the mixture was diluted with EtOAc, filtered through Celite and concentrated *in vacuo*. The crude product was purified by flash column chromatography to produce the desired product.

### 4.14. Preparation and Characterisation of Final Compounds and Intermediates

#### 4.14.1. Methyl 4-bromo-2-((cyclopropylmethyl)amino)benzoate (**2**)

General procedure A; yield 80% (989 mg, 3.48 mmol); brown oil. ^1^H NMR (400 MHz, CDCl_3_) δ 7.86 (s, 1H), 7.73 (d, *J* = 8.5 Hz, 1H), 6.79 (d, *J* = 1.9 Hz, 1H), 6.68 (dd, *J* = 8.6, 1.8 Hz, 1H), 3.85 (s, 3H), 3.01 (d, *J* = 6.9 Hz, 2H), 1.19–1.09 (m, 1H), 0.65–0.56 (m, 2H), 0.28 (dt, *J* = 5.9, 4.6 Hz, 2H); ^13^C NMR (100 MHz, CDCl_3_) δ 169.1, 152.2, 133.3, 130.2, 118.0, 114.4, 109.1, 52.0, 48.3, 10.9, 4.1; *m*/*z* (ESI^+^) 284.0 ([M+H]^+^, 100%); HRMS (ESI^+^) C_12_H_15_NO_2_Br^+^ ([M+H]^+^) requires 284.0281; found 284.0282.

#### 4.14.2. (4-Bromo-2-((cyclopropylmethyl)amino)phenyl)methanol (**3**)

General procedure B from **2** (950 mg, 3.43 mmol); yield quant. (863 mg, 3.37 mmol); light brown solid. ^1^H NMR (400 MHz, CDCl_3_) δ 6.88 (dd, *J* = 7.6, 1.9 Hz, 1H), 6.79–6.70 (m, 2H), 4.60 (s, 2H), 2.94 (dd, *J* = 6.9, 1.9 Hz, 2H), 1.20–1.04 (m, 1H), 0.62–0.53 (m, 2H), 0.32–0.20 (m, 2H), N*H* and O*H* not visible; ^13^C NMR (100 MHz, CDCl_3_) δ 149.0, 130.3, 123.7, 123.0, 119.0, 113.6, 64.3, 48.7, 10.8, 3.7; *m*/*z* (ESI^+^) 256.0 ([M+H]^+^, 100%); HRMS (ESI^+^) C_11_H_15_NOBr^+^ ([M+H]^+^) requires 256.03315; found 256.03331.

#### 4.14.3. 8-Bromo-1-(cyclopropylmethyl)-1,5-dihydrobenzo[*e*][1,4]oxazepin-2(3*H*)-one (**4**)

General procedure C.1 from **3** (840 mg, 3.28 mmol); yield 49% (472 mg, 1.59 mmol); light yellow solid. ^1^H NMR (400 MHz, CDCl_3_) δ 7.46 (d, *J* = 1.9 Hz, 1H), 7.41 (dd, *J* = 8.0, 1.9 Hz, 1H), 7.24 (d, *J* = 8.1 Hz, 1H), 4.65 (s, 2H), 3.97 (s, 2H), 3.80 (d, *J* = 7.2 Hz, 2H), 1.11–0.98 (m, 1H), 0.48–0.41 (m, 2H), 0.31–0.22 (m, 2H); ^13^C NMR (100 MHz, CDCl_3_) δ 168.0, 144.1, 131.9, 129.8, 128.9, 125.2, 123.6, 67.5, 67.4, 51.6, 10.3, 4.1; *m*/*z* (ESI^+^) 296.0 ([M+H]^+^, 70%); HRMS (ESI^+^) C_13_H_15_NO_2_Br^+^ ([M+H]^+^) requires 296.02807; found 296.02819.

#### 4.14.4. *N*-(3-(1-(Cyclopropylmethyl)-2-oxo-1,2,3,5-tetrahydrobenzo[*e*][1,4]oxazepin-8-yl)phenyl)methanesulfonamide (OXS003976 **5**)

General procedure D from **4** (50 mg, 0.17 mmol); yield 84% (55 mg, 0.14 mmol); white solid. ^1^H NMR (400 MHz, CDCl_3_) δ 7.49 (qd, *J* = 3.5, 1.9 Hz, 4H), 7.46–7.43 (m, 1H), 7.41 (dt, *J* = 7.8, 1.4 Hz, 1H), 7.29–7.24 (m, 1H), 6.94 (s, 1H), 4.75 (s, 2H), 4.02 (s, 2H), 3.89 (d, *J* = 7.2 Hz, 2H), 3.08 (s, 3H), 1.16–1.04 (m, 1H), 0.49–0.42 (m, 2H), 0.32–0.24 (m, 2H); ^13^C NMR (100 MHz, CDCl_3_) δ 168.3, 143.4, 142.3, 141.8, 137.7, 131.1, 130.5, 129.5, 125.5, 124.2, 120.6, 120.1, 119.5, 67.6, 67.6, 51.7, 39.8, 10.4, 4.1; *m*/*z* (ESI^+^) 387.1 ([M+H]^+^, 70%); HRMS (ESI^+^) C_20_H_23_N_2_O_4_S^+^ ([M+H]^+^) requires 387.13730; found 387.13728.

**Scheme 4:**
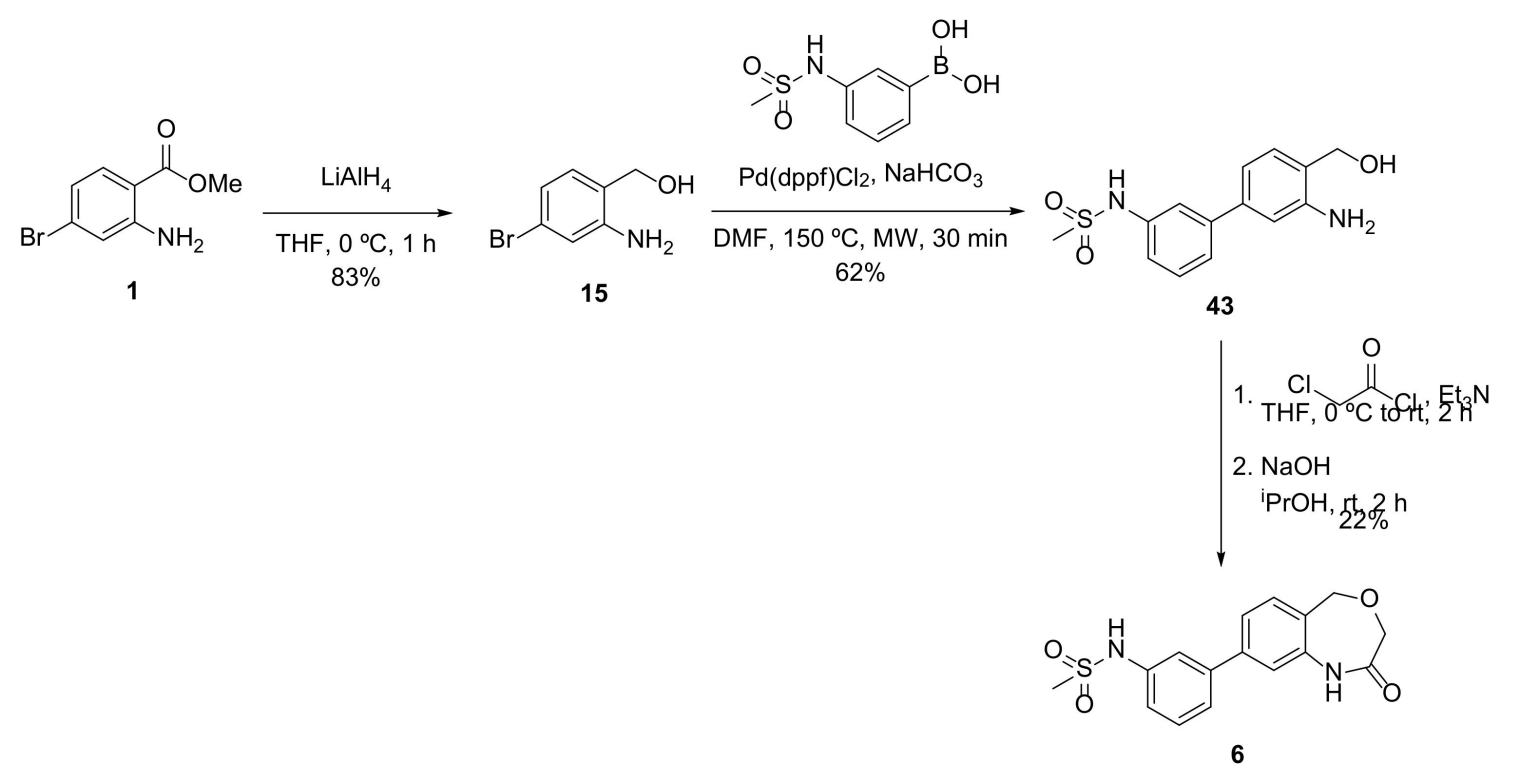
Synthesis of analogue **6**.

Compound **6** was prepared as shown in Scheme 4.

#### 4.14.5. (2-Amino-4-bromophenyl)methanol (**15**)

General procedure B from methyl 2-amino-4-bromobenzoate (500 mg, 2.17 mmol); yield 83% (363 mg, 1.80 mmol); orange solid. ^1^H NMR (400 MHz, CD_3_OD) δ 6.97 (d, *J* = 8.0 Hz, 1H), 6.89 (d, *J* = 2.0 Hz, 1H), 6.75 (dd, *J* = 8.0, 2.0 Hz, 1H), 4.51 (s, 2H), NH_2_ and OH not visible; ^13^C NMR (100 MHz, CD_3_OD) δ 149.2, 131.1, 125.6, 123.0, 121.1, 119.1, 62.9; HRMS (ESI^-^) C_7_H_7_NOBr^-^ ([M-H]^−^) requires 199.97165; found 199.97135.

#### 4.14.6. *N*-(3′-Amino-4′-(hydroxymethyl)-[1,1′-biphenyl]-3-yl)methanesulfonamide (**43**)

General procedure D from **15** (100 mg, 0.495 mmol); yield 62% (90 mg, 0.31 mmol); yellow solid. ^1^H NMR (500 MHz, CD_3_OD) δ 7.45 (t, *J* = 2.0 Hz, 1H), 7.35 (q, *J* = 7.6 Hz, 1H), 7.32 (dt, *J* = 7.7, 1.6 Hz, 1H), 7.21–7.18 (m, 1H), 7.17 (d, *J* = 7.9 Hz, 1H), 7.01 (d, *J* = 1.9 Hz, 1H), 6.92 (dd, *J* = 7.7, 1.8 Hz, 1H), 4.62 (s, 2H), 2.96 (s, 3H); ^13^C NMR (125 MHz, CD_3_OD) δ 146.4, 142.6, 141.0, 139.6, 129.2, 128.9, 125.0, 122.2, 118.9, 118.7, 116.3, 114.2, 61.9, 37.7; *m*/*z* (ESI^+^) 293.1 ([M+H]^+^, 15%); HRMS (ESI^+^) C_14_H_17_N_2_O_3_S^+^ ([M+H]^+^) requires 293.09544; found 293.09542.

#### 4.14.7. *N*-(3-(2-Oxo-1,2,3,5-tetrahydrobenzo[*e*][1,4]oxazepin-8-yl)phenyl)methanesulfonamide (**6**)

General procedure C.1 from **43** (79 mg, 0.27 mmol); yield 22% (20 mg, 0.060 mmol); white solid. ^1^H NMR (500 MHz, CDCl_3_) δ 7.86 (d, *J* = 1.9 Hz, 1H), 7.51 (d, *J* = 8.0 Hz, 1H), 7.45—7.40 (m, 3H), 7.35 (d, *J* = 7.8 Hz, 1H), 7.22 (ddd, *J* = 7.8, 2.2, 1.0 Hz, 1H), 5.48 (t, *J* = 5.4 Hz, 1H), 4.56 (d, *J* = 5.2 Hz, 2H), 4.37 (s, 2H), 3.01 (s, 3H), 1 N*H* is not visible; ^13^C NMR (125 MHz, CDCl_3_) δ 165.0, 140.8, 139.3, 138.8, 135.5, 133.9, 129.9, 128.2, 123.4, 122.0, 121.7, 118.8, 117.8, 60.1, 43.2, 39.4; *m*/*z* (ESI^−^) 331.1 ([M-H]^−^, 12%); HRMS (ESI^−^) C_16_H_15_N_2_O_4_S^-^ ([M-H]^−^) requires 331.07580; found 331.07550.

#### 4.14.8. 2-Bromo-*N*-(5-bromo-2-(hydroxymethyl)phenyl)acetamide (**44**)

To a mixture of **15** (1.0 g, 4.9 mmol) in DCM (40 mL) was added 2-bromoacetyl bromide (0.47 mL, 5.4 mmol) at 0 °C. After stirring for 5 min at 0 °C, 2 M aqueous Na_2_CO_3_ (2.5 mL) was added over a period of 10 min. The cooling bath was removed and stirring was continued for 3 h. The reaction mixture was concentrated under vacuum and the aqueous residue was extracted with EtOAc. The combined organic layers were washed with brine, dried and concentrated under vacuum. Purification by recrystallisation using a mixture of EtOAC and pentane produced the title compound **44** (1.06 g, 3.28 mmol, 67%) as an off white solid. ^1^H NMR (500 MHz, CDCl_3_ 9.53 (brs, 1H), 8.33 (d, *J* = 2.0 Hz, 1H), 7.25 (dd, *J* = 8.0, 2.0 Hz, 1H), 7.07 (d, *J* = 8.1 Hz, 1H), 4.73 (s, 2H), 4.03 (s, 2H), 2.07 (brs, 1H); ^13^C NMR (125 MHz, CDCl_3_) 164.4, 138.3, 130.1, 128.3, 128.0, 125.2, 123.0, 64.0, 29.5; *m/z* (ESI^+^) 345.8 ([M+Na]^+^, 20%); HRMS (ESI^+^) C_9_H_9_Br_2_NO_2_Na^+^ ([MNa]^+^) requires 345.88718; found 345.88718.

#### 4.14.9. 8-Bromo-1,5-dihydrobenzo[*e*][1,4]oxazepin-2(3*H*)-one (**16**)

To a suspension of **44** (0.90 g, 2.8 mmol) in 2-propanol (28 mL) was added, portion-wise, potassium *tert*-butoxide (0.81 g, 7.2 mmol) at 0 °C. The reaction mixture was stirred for 3 h and subsequently poured on ice/water. The resulting precipitate was collected by filtration, washed with water, dissolved in MeOH, dried, filtered and concentrated under vacuum to produce the cyclised product **16** (0.61 g, 2.5 mmol, 90%) as a light-yellow solid. ^1^H NMR (500 MHz, CDCl_3_) 8.83 (brs, 1H), 7.18 (dd, *J* = 8.1, 1.8 Hz, 1H), 7.11 (d, *J* = 1.8 Hz, 1H), 6.99 (d, *J* = 8.1 Hz, 1H), 4.69 (s, 2H), 4.59 (s, 2H); ^13^C NMR (125 MHz, CDCl_3_) 173.4, 137.3, 130.0, 127.8, 126.7, 122.5, 122.2, 73.7, 72.6; HRMS (ESI^+^) C_9_H_10_BrNO_2_^+^ ([MH]^+^) requires 241.98112; found 241.98129.

#### 4.14.10. 8-Bromo-1-methyl-1,5-dihydrobenzo[*e*][1,4]oxazepin-2(3*H*)-one (**17**)

To a solution of amide **16** (50 mg, 0.21 mmol) in anhydrous DMF (1 mL) were added iodomethane (25 μL, 0.41 mmol) and sodium hydride (12 mg, 0.31 mmol) at 0 °C. The mixture was stirred at room temperature overnight then quenched with saturated NH_4_Cl, extracted with 3xEtOAc, dried over anhydrous Na_2_SO_4_ and concentrated *in vacuo* to produce methylated **17** (53 mg, 0.21 mmol, quant.) as a yellow solid, which was taken to the next step without further purification. ^1^H NMR (400 MHz, CDCl_3_) δ 7.42–7.36 (m, 2H), 7.22 (d, *J* = 8.6 Hz, 1H), 4.59 (s, 2H), 4.01 (s, 2H), 3.41 (s, 3H); *m*/*z* (ESI^+^) 256.1 ([M+H]^+^, 100%); HRMS (ESI^+^) C_10_H_11_NO_2_Br^+^ ([M+H]^+^) requires 255.99677; found 255.99686.

#### 4.14.11. *N*-(3-(1-Methyl-2-oxo-1,2,3,5-tetrahydrobenzo[*e*][1,4]oxazepin-8-yl)phenyl)methanesulfonamide (**7**)

General procedure D from **17** (53 mg, 0.21 mmol); yield 28% (20 mg, 0.058 mmol); yellow solid. ^1^H NMR (400 MHz, CDCl_3_) δ 7.50 (t, *J* = 1.9 Hz, 1H), 7.48–7.37 (m, 6H), 7.29 (ddd, *J* = 7.9, 2.2, 1.2 Hz, 1H), 4.68 (s, 2H), 4.05 (s, 2H), 3.49 (s, 3H), 3.06 (s, 3H); ^13^C NMR (100 MHz, CDCl_3_) δ 168.7, 144.4, 142.5, 141.8, 137.6, 131.1, 130.5, 128.5, 125.3, 124.3, 120.2, 119.6, 119.5, 67.7, 67.7, 39.8, 34.9; *m*/*z* (ESI^+^) 347.1 ([M+H]^+^, 100%); HRMS (ESI^+^) C_17_H_19_N_2_O_4_S^+^ ([M+H]^+^) requires 347.10600; found 347.10634.

#### 4.14.12. 8-Bromo-1-ethyl-1,5-dihydrobenzo[*e*][1,4]oxazepin-2(3*H*)-one (**18**)

To a solution of amide **16** (64 mg, 0.26 mmol) in anhydrous DMF (1.3 mL) were added iodoethane (40 μL, 0.53 mmol) and sodium hydride (16 mg, 0.40 mmol) at 0 °C. The mixture was stirred at room temperature overnight, then quenched with saturated NH_4_Cl, extracted with 3xEtOAc, dried over anhydrous Na_2_SO_4_ and concentrated *in vacuo*. The crude product was purified by flash column chromatography (5% to 20% EtOAc in pentane) to produce ethyl-substituted **18** (27 mg, 0.10 mmol, 38%) as a white solid. ^1^H NMR (400 MHz, CDCl_3_) δ 7.44—7.37 (m, 2H), 7.23 (d, *J* = 8.0 Hz, 1H), 4.57 (s, 2H), 3.96 (s, 2H), 3.95 (q, *J* = 7.1 Hz, 2H), 1.22 (t, *J* = 7.1 Hz, 3H); *m/z* (ESI^+^) 270.0 ([M+H]^+^, 100%); HRMS (ESI^+^) C_11_H_13_NO_2_Br^+^ ([M+Na]^+^) requires 270.01242; found 270.01266.

#### 4.14.13. *N*-(3-(1-Ethyl-2-oxo-1,2,3,5-tetrahydrobenzo[*e*][1,4]oxazepin-8-yl)phenyl)methanesulfonamide (**8**)

General procedure D from **18** (26 mg, 0.096 mmol); yield 84% (29 mg, 0.081 mmol); yellow solid. ^1^H NMR (400 MHz, CDCl_3_) δ 7.50–7.43 (m, 5H), 7.41 (ddd, *J* = 7.8, 1.9, 0.9 Hz, 1H), 7.29–7.27 (m, 1H), 6.97 (s, 1H), 4.67 (s, 2H), 4.06 (q, *J* = 7.1 Hz, 2H), 4.02 (s, 2H), 3.07 (s, 3H), 1.26 (t, *J* = 7.1 Hz, 3H); ^13^C NMR (100 MHz, CDCl3) δ 168.0, 143.1, 142.5, 141.8, 137.7, 131.2, 130.5, 129.3, 125.5, 124.3, 120.1, 120.1, 119.5, 67.8, 67.6, 42.7, 39.8, 13.71; *m*/*z* (ESI^+^) 361.1 ([M+H]^+^, 100%); HRMS (ESI^+^) C_18_H_21_N_2_O_4_S^+^ ([M+H]^+^) requires 361.12165; found 361.12165.

#### 4.14.14. 8-Bromo-1-isopropyl-1,5-dihydrobenzo[*e*][1,4]oxazepin-2(3*H*)-one (**19**)

To a suspension of **16** (100 mg, 0.413 mmol) in dry DMF (2.8 mL) was added NaH (60% *w*/*w*, 20 mg, 0.50 mmol) at 0 °C under an inert atmosphere. After the reaction mixture was stirred at 0 °C for 30 min, 2-bromopropane (58 µL, 0.62 mmol) was added. The reaction was stirred at 50 °C for 18 h and then quenched with water at 0 °C. The aqueous layer was extracted with EtOAc and the combined organic layers were washed with brine, dried and concentrated under vacuum. The residue was purified by flash column chromatography (15–20% EtOAc in pentane) to produce the desired product **19** (66 mg, 0.23 mmol, 56%) as a white solid. ^1^H NMR (400 MHz, CDCl_3_) δ 7.42 (dt, *J* = 4.5, 2.0 Hz, 2H), 7.22 (d, *J* = 8.5 Hz, 1H), 4.64 (hept, *J* = 7.0 Hz, 1H), 4.56 (s, 2H), 3.88 (s, 2H), 1.38 (d, *J* = 7.0 Hz, 6H); ^13^C NMR (100 MHz, CDCl_3_) 167.7, 143.4, 131.7, 130.2, 129.2, 126.3, 123.1, 68.1, 67.2, 51.1, 21.3; *m*/*z* (ESI^+^) 347.1 ([M+H]^+^, 100%); HRMS (ESI^+^) C_17_H_19_N_2_O_4_S^+^ ([M+H]^+^) requires 347.10600; found 347.10634.

#### 4.14.15. *N*-(3-(1-Isopropyl-2-oxo-1,2,3,5-tetrahydrobenzo[*e*][1,4]oxazepin-8-yl)phenyl)methanesulfonamide (OXS007002 **9**)

General procedure D from **19** (20 mg, 0.070 mmol); yield 91% (24 mg, 0.064 mmol); yellow solid. ^1^H NMR (500 MHz, CDCl_3_) δ 7.53–7.46 (m, 2H), 7.48–7.43 (m, 2H), 7.46–7.38 (m, 2H), 7.24 (ddd, *J* = 7.9, 2.4, 1.0 Hz, 1H), 6.47 (s, 1H), 4.75 (hept, *J* = 6.9 Hz, 1H), 4.67 (s, 2H), 3.95 (s, 2H), 3.07 (s, 3H), 1.44 (d, *J* = 6.9 Hz, 6H); ^13^C NMR (125 MHz, CDCl_3_) δ 168.0, 142.6, 141.9, 141.9, 137.5, 131.0, 130.6, 129.9, 125.9, 124.3, 121.9, 120.1, 119.5, 68.2, 67.5, 50.8, 39.9, 21.6, 1.2; *m*/*z* (ESI^+^) 375.1 ([M+H]^+^, 100%); HRMS (ESI^+^) C_19_H_23_N_2_O_4_S^+^ ([M+H]^+^) requires 375.13730; found 375.13681.

#### 4.14.16. 8-Bromo-1-isobutyl-1,5-dihydrobenzo[*e*][1,4]oxazepin-2(3*H*)-one (**20**)

To a suspension of **16** (0.30 g, 1.2 mmol) in dry DMF (8.3 mL) were added 1-iodo-2-methylpropane (0.29 mL, 2.5 mmol) and Cs_2_CO_3_ (0.81 g, 2.48 mmol). The reaction was stirred at room temperature for 48 h and then quenched with water. The aqueous layer was extracted with EtOAc and the combined organic layers were washed with brine, dried and concentrated under vacuum. The residue was purified by flash column chromatography (25–30% EtOAc in pentane), to produce the desired compound **20** (0.24 g, 0.80 mmol, 64%) as a white solid. ^1^H NMR (500 MHz, CDCl_3_) 7.41 (d, *J* = 1.9 Hz, 1H), 7.39 (dd, *J* = 7.9 Hz, 1.9, 1H), 7.23 (d, *J* = 8.0 Hz, 1H), 4.65 (s, 2H), 3.97 (s, 2H), 3.77 (d, *J* = 7.5 Hz, 2H), 1.94 (tsep, *J* = 13.7, 6.9 Hz, 1H), 0.88 (d, *J* = 6.5 Hz, 6H); ^13^C NMR (125 MHz, CDCl_3_) 168.3, 144.1, 132.2, 129.5, 128.4, 124.4, 123.7, 67.8, 67.7, 53.7, 27.4, 20.6; *m*/*z* (ESI^+^) 298.1 ([M+H]^+^, 100%); HRMS (ESI^+^) C_13_H_17_BrNO_2_^+^ ([M+H]^+^) requires 298.04372; found 298.04383.

#### 4.14.17. *N*-(3-(1-Isobutyl-2-oxo-1,2,3,5-tetrahydrobenzo[*e*][1,4]oxazepin-8-yl)phenyl)methanesulfonamide (**10**)

General procedure D from **20** (30 mg, 0.10 mmol; yield 62% (24 mg, 0.062 mmol); white solid. ^1^H NMR (500 MHz, CDCl_3_) 7.50–7.37 (m, 6H), 7.24 (ddd, *J* = 8.0, 2.3, 1.1 Hz, 1H) 6.64 (s, 1H), 4.47 (s, 2H), 4.03 (s, 2H), 3.87 (d, *J* = 7.3 Hz, 2H), 3.07 (s, 3H), 1.99 (tsep, *J* = 13.7, 6.8 Hz, 1H), 0.90 (d, *J* = 6.7 Hz, 6H); ^13^C NMR (125 MHz, CDCl_3_) 168.6, 143.4, 142.3, 141.9, 137.6, 131.5, 130.5, 129.0, 125.3, 124.3, 120.1, 119.9, 119.5, 67.9 (2×C), 53.8, 39.9, 27.5, 20.7; *m/z* (ESI^+^) 389.1 ([M+H]^+^, 100%); HRMS (ESI^+^) C_20_H_25_N_2_O_4_S^+^ ([M+H]^+^) requires 389.15295; found 389.15300.

#### 4.14.18. 8-Bromo-1-(2,2,2-trifluoroethyl)-1,5-dihydrobenzo[*e*][1,4]oxazepin-2(3*H*)-one (**21**)

To a suspension of **16** (0.20 g, 0.83 mmol) in dry DMF (5.5 mL) was added NaH (60% w/w in mineral oil, 40 mg, 0.99 mmol) at 0 °C under an inert atmosphere. After the reaction mixture was stirred at 0 °C for 30 min, 2,2,2-trifluoroethyl trifluoromethanesulfonate (0.23 g, 0.99 mmol) was added. The reaction was stirred at room temperature for 18 h and then quenched with water at 0 °C. The aqueous layer was extracted with EtOAc and the combined organic layers were washed with brine, dried and concentrated under vacuum. The residue was purified by flash column chromatography (10% EtOAc in pentane) to produce the desired product **21** (91 mg, 0.28 mmol, 34%) as a white solid. ^1^H NMR (500 MHz, CDCl_3_) 7.50 (dd, *J* = 8.1, 1.8 Hz, 1H), 7.46 (d, *J* = 1.8 Hz, 1H), 7.30 (d, *J* = 8.1 Hz, 1H), 4.68 (s, 2H), 4.59 (q, *J* = 8.5 Hz, 2H), 4.04 (s, 2H); ^13^C NMR (125 MHz, CDCl_3_) 168.1, 142.4, 132.3, 130.8, 128.6, 127.1, 124.9, 124.3, 123.8, 122.7, 120.4, 67.51, 66.5, 46.9, 46.7, 46.4, 46.1; *m*/*z* (ESI^+^) 323.9 ([M+H]^+^, 100%); HRMS (ESI^+^) C_11_H_10_BrF_3_NO_2_^+^ ([M+H]^+^) requires 323.98415; found 323.98420.

#### 4.14.19. *N*-(3-(2-Oxo-1-(2,2,2-trifluoroethyl)-1,2,3,5-tetrahydrobenzo[*e*][1,4]oxazepin-8-yl)phenyl)methanesulfonamide (**11**)

General procedure D from **21** (30 mg, 0.10 mmol); yield 70% (28 mg, 0.068 mmol); white solid. ^1^H NMR (500 MHz, CDCl_3_) δ 7.53 (dd, *J* = 7.8, 1.7 Hz, 1H), 7.46 (ddd, *J* = 7.9, 3.4, 1.6 Hz, 4H), 7.39 (dt, *J* = 7.8, 1.3 Hz, 1H), 7.28–7.25 (m, 1H), 4.73 (s, 2H), 4.66 (q, *J* = 8.6 Hz, 2H), 4.06 (s, 2H), 3.07 (s, 3H), N*H* not visible; ^13^C NMR (125 MHz, CDCl_3_) δ 168.7, 142.9, 142.0, 141.5, 138.0, 131.7, 130.7, 129.2, 126.6, 124.3, 124.2 (q, *J* = 281 Hz), 120.4, 119.9, 119.6, 67.5, 66.8, 46.9 (q, *J* = 34 Hz), 39.8; *m*/*z* (ESI^+^) 415.0 ([M+H]^+^, 100%); HRMS (ESI^+^) C_18_H_18_F_3_N_2_O_4_S ^+^ ([M+H]^+^) requires 415.09339; found 415.09328.

#### 4.14.20. 8-Bromo-1-cyclopentyl-1,5-dihydrobenzo[*e*][1,4]oxazepin-2(3*H*)-one (**22**)

To a solution of **16** (100 mg, 0.41 mmol) in DMF (2 mL) were added Cs_2_CO_3_ (404 mg, 1.24 mmol) and bromocyclopentane (0.13 mL, 1.24 mmol). The mixture was stirred at 50 °C for 16 h, cooled to room temperature, diluted with EtOAc washed with brine (3×), dried and concentrated *in vacuo*. The crude product was purified by flash column chromatography (10–40% EtOAc in pentane) to produce **22** (42 mg, 0.14 mmol, 33%) as a yellow oil. ^1^H NMR (500 MHz, CDCl_3_) δ 7.45 (dd, *J* = 8.0, 1.9 Hz, 1H), 7.42 (d, *J* = 1.9 Hz, 1H), 7.24 (d, *J* = 8.0 Hz, 1H), 4.61 (s, 2H), 4.49 (p, *J* = 8.8 Hz, 1H), 3.91 (s, 2H), 2.05–1.96 (m, 4H), 1.96–1.85 (m, 2H), 1.68–1.59 (m, 2H); ^13^C NMR (125 MHz, CDCl_3_) δ 167.7, 144.4, 131.5, 129.9, 128.9, 126.2, 123.1, 68.1, 67.1, 61.5, 29.8 (2 × C), 24.6 (2 × C); HRMS (ESI^+^) C_14_H_17_BrNO_2_^+^ ([M+H]^+^) requires 310.0437; found 310.0438.

#### 4.14.21. *N*-(3-(1-Cyclopentyl-2-oxo-1,2,3,5-tetrahydrobenzo[*e*][1,4]oxazepin-8-yl)phenyl)methanesulfonamide (**12**)

General procedure D from **22** (30 mg, 0.10 mmol); yield 40% (16 mg, 0.04 mmol); white solid. ^1^H NMR (500 MHz, CDCl_3_) δ 7.51–7.44 (m, 3H), 7.44–7.37 (m, 3H), 7.24 (ddd, *J* = 7.4, 2.5, 1.0 Hz, 1H), 6.69 (s, 1H), 4.67 (s, 2H), 4.60 (p, *J* = 8.8 Hz, 1H), 3.94 (s, 2H), 3.07 (s, 3H), 2.06–1.97 (m, 4H), 1.92–1.84 (m, 2H), 1.65–1.59 (m, 2H); ^13^C NMR (125 MHz, CDCl_3_) δ 168.1, 143.5, 141.9, 141.7, 137.5, 130.8, 130.4, 129.5, 125.7, 124.1, 121.9, 119.9, 119.3, 68.2, 67.4, 61.2, 39.7, 30.0 (2 × C), 24.7 (2 × C); HRMS (ESI^+^) C_21_H_23_N_2_O_4_S^+^ ([M+H]^+^) requires 401.1530; found 401.1530.

#### 4.14.22. 8-Bromo-1-(oxetan-3-yl)-1,5-dihydrobenzo[*e*][1,4]oxazepin-2(3*H*)-one (**23**)

To a suspension of **16** (100 mg, 0.41 mmol) in dry DMF (2.7 mL) were added Cs_2_CO_3_ (269 mg, 0.830 mmol) and 3-bromooxetane (51 µL, 0.62 mmol). The reaction was stirred at 50 °C for 3 days and then quenched with water. The aqueous layer was extracted with EtOAc and the combined organic layers were washed with brine, dried and concentrated under vacuum. The residue was purified by flash column chromatography (10–25% EtOAc in pentane) to produce the desired product **23** (54 mg, 0.18 mmol, 44%) which was used in the next step without additional purification. ^1^H NMR (400 MHz, CDCl_3_) δ 7.43 (dd, *J* = 8.1, 1.8 Hz, 1H), 7.29 (d, *J* = 8.1 Hz, 1H), 6.90 (d, *J* = 1.8 Hz, 1H), 5.20 (p, *J* = 7.3 Hz, 1H), 4.89 (t, *J* = 7.5 Hz, 2H), 4.76 (s, 2H), 4.55 (t, *J* = 7.4 Hz, 2H), 3.97 (s, 2H); *m*/*z* (ESI^+^) 297.9 ([M+H]^+^, 100%).

#### 4.14.23. *N*-(3-(1-(Oxetan-3-yl)-2-oxo-1,2,3,5-tetrahydrobenzo[*e*][1,4]oxazepin-8-yl)phenyl)methanesulfonamide (**13**)

General procedure D from **23** (30 mg, 0.10 mmol); yield 20% (7.7 mg, 0.020 mmol); white solid. ^1^H NMR (500 MHz, CDCl_3_) 7.50–7.45 (m, 3H), 7.42 (d, *J* = 2.0 Hz, 1H), 7.34 (d, *J* = 7.8 Hz, 1H), 7.22 (dd, *J* = 7.9, 2.3 Hz, 1H), 6.92 (s, 1H), 5.32 (p, *J* = 7.2 Hz, 1H), 4.92 (t, *J* = 7.2 Hz, 2H), 4.84 (s, 2H), 4.61 (t, *J* = 7.1 Hz, 2H), 4.02 (s, 2H), 3.07 (s, 3H), N*H* not visible; ^13^C NMR (125 MHz, CDCl_3_) 168.2, 142.4, 141.4, 140.8, 137.6, 131.5, 130.6, 129.2, 125.7, 124.2, 120.1, 119.3, 119.1, 75.7, 67.9, 67.5, 51.7, 39.9; *m*/*z* (ESI^+^) 389.1 ([M+H]^+^, 100%); HRMS (ESI^+^) C_19_H_21_N_2_O_5_S^+^ ([M+H]^+^) requires 389.11657; found 389.11688.

#### 4.14.24. 8-Bromo-1-phenyl-1,5-dihydrobenzo[*e*][1,4]oxazepin-2(3*H*)-one (**24**)

To a solution of **16** (100 mg, 0.41 mmol) in THF (2.8 mL) and DCM (2.8 mL) was added Et_3_N (0.43 mL, 3.07 mmol). This was followed by the addition of Cu(OAc)_2_·H_2_O (131 mg, 0.66 mmol) and phenylboronic acid (101 mg, 0.83 mmol). The reaction mixture was stirred at room temperature for 41 h. Upon completion, it was filtrated through Celite and concentrated under vacuum. The crude was purified by flash column chromatography (15–20% EtOAc in pentane) to produce **24** as an off-white solid (65 mg, 0.20 mmol, 50%). ^1^H NMR (500 MHz, CDCl_3_) 7.46–7.42 (m, 2H), 7.40 (dd, *J* = 8.1, 1.9 Hz, 1H), 7.38–7.33 (m, 1H), 7.29 (d, *J* = 8.1 Hz, 1H), 7.22–7.17 (m, 2H), 7.01 (d, *J* = 1.9 Hz, 1H), 4.80 (s, 2H), 4.16 (s, 2H); ^13^C NMR (125 MHz, CDCl_3_) 167.9, 144.9, 140.3, 131.9, 129.9, 129.7, 128.3, 128.0, 127.8, 126.8, 123.6, 68.2, 67.7; *m/z* (ESI^+^) 318.0 ([M+H]^+^, 100%); HRMS (ESI^+^) C_15_H_13_BrNO_2_^+^ ([M+H]^+^) requires 318.01242; found 318.01260.

#### 4.14.25. *N*-(3-(2-Oxo-1-phenyl-1,2,3,5-tetrahydrobenzo[*e*][1,4]oxazepin-8-yl)phenyl)methanesulfonamide (**14**)

General procedure D from **24** (30 mg, 0.09 mmol); yield 75% (29 mg, 0.071 mmol); white solid. ^1^H NMR (500 MHz, CDCl_3_) 7.51–7.32 (m, 7H), 7.23–7.17 (m, 4H), 7.02 (d, *J* = 1.7 Hz, 1H), 6.43 (brs, 1H), 4,81 (s, 2H), 4.21 (s, 2H), 3.01 (s, 3H); ^13^C NMR (125 MHz, CDCl_3_) 168.2, 144.2, 142.3, 141.6, 140.8, 137.4, 131.1, 130.4, 129.5, 129.0, 127.8, 127.7, 125.7, 124.3, 122.5, 119.9, 119.4, 68.3, 67.9, 39.8; *m/z* (ESI^+^) 409.1 ([M+H]^+^, 100%); HRMS (ESI^+^) C_22_H_21_N_2_O_4_S^+^ ([M+H]^+^) requires 409.12165; found 409.12159.

#### 4.14.26. *N*-(2-(1-Isopropyl-2-oxo-1,2,3,5-tetrahydrobenzo[*e*][1,4]oxazepin-8-yl)phenyl)methanesulfonamide (**25**)

General procedure D from **19** (20 mg, 0.070 mmol); yield 87% (23 mg, 0.061 mmol); a yellow solid. ^1^H NMR (700 MHz, CDCl_3_) 7.66 (d, *J* = 8.2 Hz, 1H), 7.54–7.45 (m, 2H), 7.32–7.30 (m, 4H), 6.38 (s, 1H), 4.70–4.65 (m, 3H), 3.99 (s, 2H), 3.03 (s, 3H), 1.45 (d, *J* = 6.9 Hz, 6H); ^13^C NMR (176 MHz, CDCl_3_) 167.9, 143.4, 139.3, 134.1, 132.0, 131.5, 131.0, 130.4, 129.8, 127.6, 125.2, 123.9, 120.0, 68.4, 67.5, 51.5, 40.6, 21.5; *m/z* (ESI^+^) 375.1 ([M+H]^+^, 100%); HRMS (ESI^+^) C_19_H_23_N_2_O_4_S^+^ ([M+H]^+^) requires 375.13730; found 375.13744.

#### 4.14.27. *N*-(4-(1-Isopropyl-2-oxo-1,2,3,5-tetrahydrobenzo[*e*][1,4]oxazepin-8-yl)phenyl)methanesulfonamide (**26**)

General procedure D from **19** (30 mg, 0.11 mmol); yield 88% (35 mg, 0.093 mmol); white solid. ^1^H NMR (500 MHz, CDCl_3_) 7.60–7.56 (m, 2H), 7.48 (dd, *J* = 7.7, 1.8 Hz, 1H), 7.44–7.40 (m, 2H), 7.36–7.32 (m, 2H), 6.62 (s, 1H), 4.75 (h, *J* = 6.9 Hz, 1H), 4.66 (s, 2H), 3.94 (s, 2H), 3.08 (s, 3H), 1.43 (d, *J* = 6.9 Hz, 6H); ^13^C NMR (125 MHz, CDCl_3_) 168.1, 142.6, 141.9, 137.2, 136.8, 131.0, 129.5, 128.6, 125.6, 121.6, 121.2, 68.2, 67.5, 50.8, 39.8, 21.6; *m/z* (ESI^+^) 375.1 ([M+H]^+^, 100%); HRMS (ESI^+^) C_19_H_23_N_2_O_4_S^+^ ([M+H]^+^) requires 375.13730; found 375.13742.

**Scheme 5:**
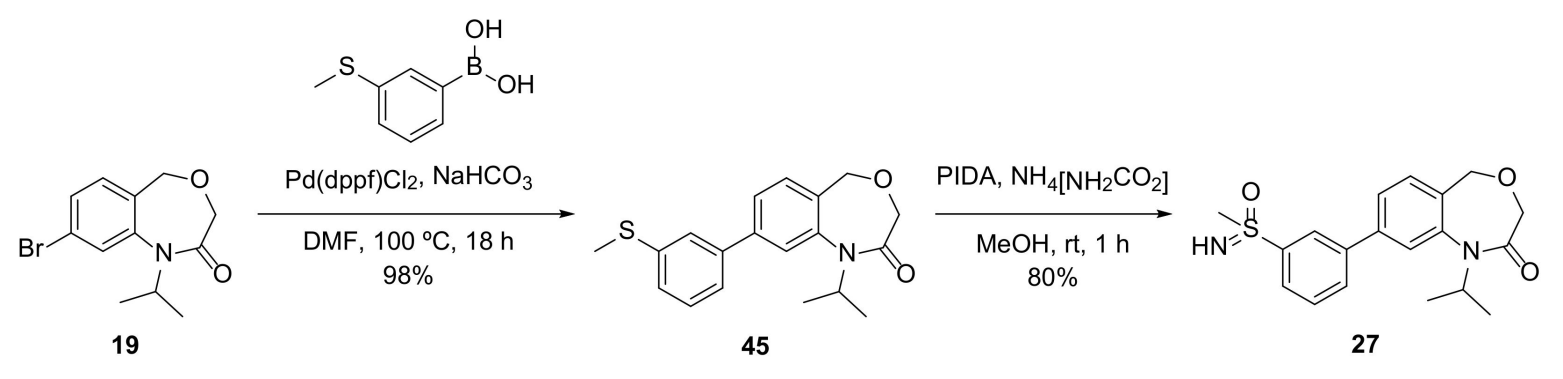
Synthesis of analogue **27**.

Compound **27** was prepared as shown in Scheme 5.

#### 4.14.28. 1-Isopropyl-8-(3-(methylthio)phenyl)-1,5-dihydrobenzo[*e*][1,4]oxazepin-2(3*H*)-one (**45**)

General procedure D from **19** (80 mg, 0.28 mmol); yield 98% (91 mg, 0.28 mmol); pale yellow solid. ^1^H NMR (500 MHz, CDCl_3_) 7.42 (dd, *J* = 7.8, 1.7 Hz, 1H), 7.38–7.32 (m, 4H), 7.27 (ddd, *J* = 7.7, 1.8, 1.2 Hz, 1H), 7.22 (ddd, *J* = 7.7, 1.9, 1.2 Hz, 1H), 4.67 (h, *J* = 6.9 Hz, 1H), 4.59 (s, 2H), 3.87 (s, 2H), 2.48 (s, 3H), 1.36 (d, *J* = 6.9 Hz, 6H); ^13^C NMR (125 MHz, CDCl_3_) 168.0, 142.6, 142.4, 140.7, 139.6, 130.9, 129.6, 129.5, 126.0, 125.9, 125.4, 124.1, 121.9, 68.2, 67.5, 50.8, 21.6, 16.0; *m*/*z* (ESI^+^) 328.1 ([M+H]^+^, 100%); HRMS (ESI^+^) C_19_H_22_NO_2_S^+^ ([M+H]^+^) requires 328.13767; found 328.13655.

#### 4.14.29. 1-Isopropyl-8-(3-(S-methylsulfonimidoyl)phenyl)-1,5-dihydrobenzo[*e*][1,4]oxazepin-2(3*H*)-one (27)

To a solution of **45** (30 mg, 0.12 mmol) in MeOH (0.25 mL) were added (diacetoxyiodo)benzene (74 mg, 0.23 mmol) and ammonium carbamate (14 mg, 0.23 mmol). The reaction was stirred at room temperature for 1 h. Upon completion the reaction was concentrated under vacuum and purified by flash column chromatography (0–1% MeOH in EtOAc) to produce the desired product **27** (26 mg, 0.073 mmol, 80%) as pale-yellow oil. ^1^H NMR (500 MHz, CDCl_3_) 8.22 (t, *J* = 1.9, 1H), 8.05 (ddd, *J* = 7.8, 1.9, 1.1 Hz, 1H), 7.82 (ddd, *J* = 7.8, 1.9, 1.1 Hz, 1H), 7.68 (t, *J* = 1.9 Hz, 1H), 7.54 (dd, *J* = 7.7, 1.8 Hz, 1H), 7.47 (s, 1H), 7.45 (d, *J* = 6, 1H), 4.72 (h, *J* = 6.9 Hz, 1H), 4.67 (s, 2H), 3.93 (s, 2H), 3.17 (s, 3H), 1.43 (d, *J* = 6.9 Hz, 6H), N*H* not visible; ^13^C NMR (125 MHz, CDCl_3_) 168.0, 144.7, 142.8, 141.5, 141.2, 131.8, 131.2, 130.3, 130.2, 127.2, 126.4, 126.1, 121.9, 68.2, 67.4, 51.1, 46.4, 21.6; *m*/*z* (ESI^+^) 359.1 ([M+H]^+^, 100%); HRMS (ESI^+^) C_19_H_23_N_2_O_3_S^+^ ([M+H]^+^) requires 359.14239; found 359.14225.

#### 4.14.30. 8-(3-Aminophenyl)-1-isopropyl-1,5-dihydrobenzo[*e*][1,4]oxazepin-2(3*H*)-one (**29**)

General procedure D from **19** (1.50 g, 5.28 mmol); yield 70% (1.10 g, 3.71 mmol); orange solid. ^1^H NMR (400 MHz, DMSO-*d*_6_) δ 7.51 (m, 2H), 7.45 (s, 1H), 7.15–7.11 (m, 1H), 6.9–6.89 (m, 1H), 6.84–6.82 (m, 1H), 6.63–6.60 (m, 1H), 5.16 (br s, 2H), 4.62–4.55 (m, 3H), 3.75 (s, 2H), 1.38 (s, 3H), 1.37 (s, 3H); ^13^C NMR (100 MHz, DMSO-*d*_6_) δ 167.6, 149.7, 143.2, 142.7, 140.3, 131.1, 130.0, 129.1, 125.4, 121.3, 114.9, 114.2, 112.7, 68.0, 66.8, 50.9, 21.5; *m/z* (ESI^+^) 297.2 ([M+H]^+^, 100%); HRMS (ESI^+^) C_18_H_21_N_2_O_2_^+^ ([M+H]^+^) requires 297.1598; found 297.1598.

#### 4.14.31. Methyl (3-(1-isopropyl-2-oxo-1,2,3,5-tetrahydrobenzo[*e*][1,4]oxazepin-8-yl)phenyl)carbamate (**28**)

A solution of amine **29** (97 mg, 0.33 mmol), triethylamine (68 μL, 0.49 mmol) and methyl chloroformate (25 μL, 0.33 mmol) in DCM (4 mL) was stirred at room temperature for 18 h. LCMS showed 75% starting material and 14% product. More methyl chloroformate (75 μL, 0.98 mmol) and triethylamine (204 μL, 1.47 mmol) were added and stirred at room temperature for 18 h. More methyl chloroformate (100 μL, 1.31 mmol) and triethylamine (204 μL, 1.47 mmol) were added and stirred at room temperature for 1.5 h. The solution was washed with 1 M HCl (6 mL), concentrated *in vacuo*, and purified by flash column chromatography (0.5% to 3% MeOH in DCM) to produce carbonate **28** (63 mg, 0.18 mmol, 54%) as an off white solid. ^1^H NMR (400 MHz, CDCl_3_) δ 7.13 (s, 1H), 7.51–7.35 (m, 5H), 7.28 (d, *J* = 7.6 Hz, 1H), 6.77 (s, 1H), 4.74 (quin, *J* = 6.8 Hz, 1H), 4.65 (s, 2H), 3.94 (s, 2H), 3.80 (s, 3H), 1.43 (d, *J* = 6.8 Hz, 6H); ^13^C NMR (100 MHz, CDCl_3_) δ 167.9, 154.0, 142.5, 142.2, 140.9, 138.6, 137.0, 129.7, 129.3, 125.8, 122.2, 121.8, 118.2, 117.4, 68.0, 67.3, 52.4, 50.7, 21.4; *m/z* (ESI^+^) 355.1 ([M+H]^+^, 100%); HRMS (ESI^+^) C_20_H_23_N_2_O_4_^+^ ([M+H]^+^) requires 355.1652; found 355.1653.

#### 4.14.32. 8-(3-(Dimethylamino)phenyl)-1-isopropyl-1,5-dihydrobenzo[*e*][1,4]oxazepin-2(3*H*)-one (**30**)

General procedure D from **19** (150 mg, 0.528 mmol); yield 60% (103 mg, 0.317 mmol); orange solid. ^1^H NMR (400 MHz, CDCl_3_) δ δ 7.51 (dd, *J* = 1.6, 7.6 Hz, 1H), 7.47 (d, *J* = 1.6 Hz, 1H), 7.39 (d, *J* = 8.0 Hz, 1H), 7.35 (t, *J* = 7.8 Hz, 1H), 6.92 (d, *J* = 7.6 Hz, 1H), 6.89 (s, 1H), 6.79 (dd, *J* = 2.4, 8.0 Hz, 1H), 4.75 (quin, *J* = 6.8 Hz, 1H), 4.66 (s, 2H), 3.95 (s, 2H), 3.03 (s, 6H), 1.43 (s, 3H), 1.42 (s, 3H); ^13^C NMR (100 MHz, CDCl_3_) δ 168.0, 151.0, 144.0, 142.1, 141.0, 130.5, 129.7, 128.9, 125.9, 122.0, 115.6, 112.2, 111.2, 68.1, 67.4, 50.6, 40.6, 21.4; *m/z* (ESI^-^) 309.1 ([M-Me]^-^, 18%); HRMS (ESI^+^) C_20_H_25_N_2_O_2_^+^ ([M+H]^+^) requires 325.1911; found 325.1911.

#### 4.14.33. 1-Isopropyl-8-(3-methoxyphenyl)-1,5-dihydrobenzo[*e*][1,4]oxazepin-2(3*H*)-one (**31**)

General procedure D from **19** (30 mg, 0.11 mmol); yield 67% (22 mg, 0.07 mmol); white solid. ^1^H NMR (500 MHz, CDCl_3_) δ 7.51 (dd, *J* = 7.8, 1.7 Hz, 1H), 7.46 (d, *J* = 1.7 Hz, 1H), 7.44–7.37 (m, 2H), 7.26 (s, 1H), 7.17 (ddd, *J* = 7.6, 1.7, 0.9 Hz, 1H), 7.11 (t, *J* = 2.1 Hz, 1H), 6.95 (ddd, *J* = 8.2, 2.6, 0.9 Hz, 1H), 4.74 (p, *J* = 6.9 Hz, 1H), 4.66 (s, 2H), 3.94 (s, 2H), 3.89 (s, 3H), 1.43 (d, *J* = 6.9 Hz, 6H); ^13^C NMR (125 MHz, CDCl_3_) δ 168.0, 160.1, 142.8, 142.3, 141.4, 130.7, 130.1, 129.3, 125.8, 121.8, 119.6, 113.3, 113.1, 68.1, 67.4, 55.4, 50.7, 21.5 (2 × C); HRMS (ESI^+^) C_19_H_22_NO_3_^+^ ([M+H]^+^) requires 312.1594; found 312.1590.

#### 4.14.34. 3-(1-Isopropyl-2-oxo-1,2,3,5-tetrahydrobenzo[*e*][1,4]oxazepin-8-yl)benzoic acid (**32**)

General procedure D from **19** (120 mg, 0.422 mmol); yield 33% (46 mg, 0.141 mmol); beige solid. ^1^H NMR (400 MHz, CDCl_3_) δ 13.03 (bs, 1H), 8.21 (t, *J* = 1.6 Hz, 1H), 7.99 (dd, *J* = 1.6, 7.6 Hz, 2H), 7.68–7.59 (m, 2H), 7.57 (d, *J* = 2.0 Hz, 2H), 4.62 (s, 2H), 4.61–4.54 (m, 1H), 3.77 (s, 2H), 1.39 (d, *J* = 6.8 Hz, 6H); ^13^C NMR (100 MHz, CDCl_3_) δ 167.6, 149.7, 143.2, 142.7, 140.3, 131.1, 130.0, 129.1, 125.4, 121.3, 114.9, 114.2, 112.7, 68.0, 66.8, 50.9, 21.5; *m/z* (ESI^−^) 324.2 ([M-H]^−^, 100%); HRMS (ESI^+^) C_19_H_20_NO_4_^+^ ([M+H]^+^) requires 326.1387; found 326.1387.

#### 4.14.35. *N*-(3-(1-Isopropyl-2-oxo-1,2,3,5-tetrahydrobenzo[*e*][1,4]oxazepin-8-yl)phenyl)propane-1-sulfonamide (**33**)

Aniline **29** (120 mg, 0.405 mmol) was stirred with triethylamine (68 μL, 0.49 mmol) in DCM (4 mL) and propanesulfonyl chloride (46 μL, 0.40 mmol). The solution was stirred at room temperature for 1 h, then washed with water, dried over anhydrous MgSO_4_ and concentrated *in vacuo*. The resulting residue was passed down a silica plug with 0.5% MeOH in DCM, then stirred overnight in pentane filtered and dried to produce sulfonamide **33** (17 mg, 0.042 mmol, 10%) as a beige solid. ^1^H NMR (400 MHz, CDCl_3_) δ 7.51–7.39 (m, 6H), 7.37–7.22 (m, 1H), 6.73 (bs, 1H), 4.76 (quin, *J* = 6.9 Hz, 1H), 4.67 (s, 2H), 3.95 (s, 2H), 3.15–3.11 (m, 2H), 1.90 (td, *J* = 15.2 Hz, 7.6 Hz, 2H), 1.44 (d, *J* = 6.8 Hz, 6H), 1.05 (t, *J* = 7.4 Hz, 3H); ^13^C NMR (100 MHz, CDCl_3_) δ 168.0, 142.4, 141.9, 141.6, 137.7, 130.9, 130.3, 129.7, 125.8, 123.8, 121.8, 119.6, 119.0, 68.1, 67.3, 53.7, 50.7, 21.5, 17.3, 12.9; *m/z* (ESI^+^) 403.1 ([M+H]^+^, 100%); HRMS (ESI^+^) C_21_H_27_N_2_O_4_S^+^ ([M+H]^+^) requires 403.1686; found 403.1687.

#### 4.14.36. 1,1,1-Trifluoro-N-(3-(1-isopropyl-2-oxo-1,2,3,5-tetrahydrobenzo[*e*][1,4]oxazepin-8-yl)phenyl)methanesulfonamide (**34**)

Aniline **29** (120 mg, 0.40 mmol) in DCM (4.0 mL) was cooled in an ice bath. Triflic anhydride (0.068 mL, 0.41 mmol) was added followed by triethylamine (0.056 mL, 0.40 mmol) and the reaction mixture was stirred at room temperature overnight. TLC showed that the reaction was not complete. The mixture was cooled and more triflic anhydride (0.068 mL, 0.41 mmol) was added. After 6 h, more triflic anhydride (0.068 mL, 0.41 mmol) was added and stirred at room temperature overnight. The reaction mixture was quenched with saturated aqueous NH_4_Cl, separated and dried with Na_2_SO_4_. The crude residue was purified by flash column chromatography (0–35% EtOAc in hexane) to produce **34** (17 mg, 0.040 mmol, 10%) as a pale orange glassy oil. ^1^H NMR (400 MHz, DMSO-*d*_6_) δ 7.65–7.51 (m, 6H), 7.32–7.30 (m, 1H), 4.64–4.57 (m, 3H), 3.77 (s, 2H), 1.38 (s, 3H), 1.37 (s, 3H); ^13^C NMR (100 MHz, DMSO-*d*_6_) δ 167.6, 143.0, 141.2, 140.9, 136.6, 131.5, 130.8, 129.9, 125.6, 125.4, 122.5, 121.9, 121.6, 121.4, 68.0, 66.8, 51.0, 21.4; *m/z* (ESI^+^) 429.1 ([M+H]^+^, 100%).

**Scheme 6:**
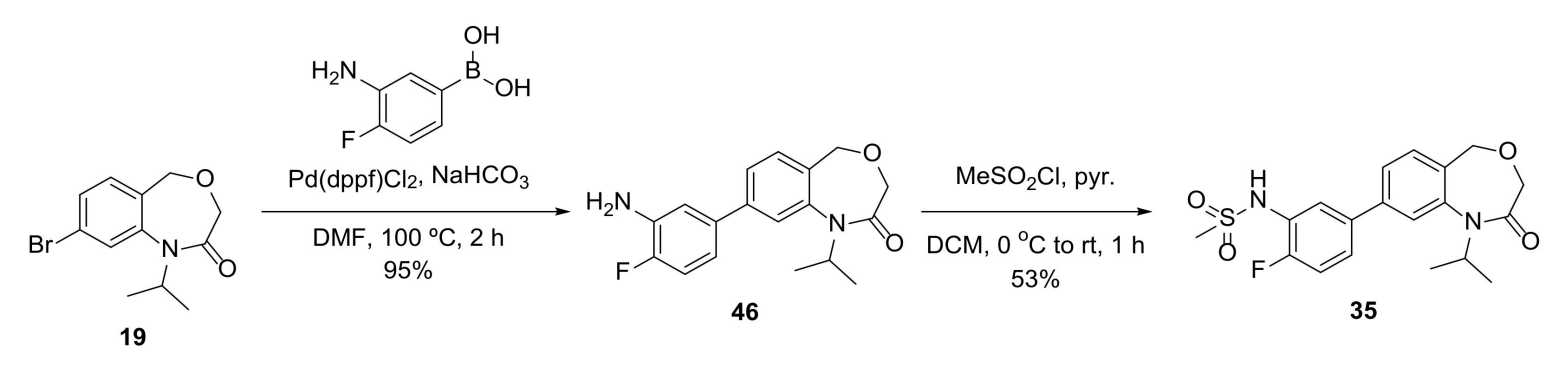
Synthesis of analogue **35**.

Compound **35** was prepared as shown in Scheme 6.

#### 4.14.37. 8-(3-Amino-4-fluorophenyl)-1-isopropyl-1,5-dihydrobenzo[*e*][1,4]oxazepin-2(3*H*)-one (**46**)

General procedure D from **19** (34 mg, 0.12 mmol); yield 95% (36 mg, 0.11 mmol); white solid. ^1^H NMR (600 MHz, CDCl_3_) δ 7.43 (dd, *J* = 7.7, 1.8 Hz, 1H), 7.40–7.36 (m, 2H), 7.07 (dd, *J* = 10.7, 8.4 Hz, 1H), 6.97 (dd, *J* = 8.4, 2.3 Hz, 1H), 6.88 (ddd, *J* = 8.4, 4.4, 2.3 Hz, 1H), 4.73 (hept, *J* = 6.9 Hz, 1H), 4.65 (s, 2H), 3.93 (s, 2H), 3.85 (s, 2H), 1.43 (d, *J* = 6.9 Hz, 6H); ^13^C NMR (151 MHz, CDCl_3_) δ 168.0, 151.7 (d, *J* = 241 Hz), 142.3 (d, *J* = 30 Hz), 136.6 (d, *J* = 3 Hz), 134.9 (d, *J* = 13 Hz), 130.7, 129.0, 125.6, 121.6, 117.4 (d, *J* = 7 Hz), 115.8, 115.7, 115.5 (d, *J* = 4 Hz), 68.1, 67.4, 50.7, 24.9, 21.5 (2 × C); HRMS (ESI^+^) C_18_H_20_FN_2_O_2_^+^ ([M+H]^+^) requires 315.1503; found 315.1502.

#### 4.14.38. *N*-(2-Fluoro-5-(1-isopropyl-2-oxo-1,2,3,5-tetrahydrobenzo[*e*][1,4]oxazepin-8-yl)phenyl)methanesulfonamide (**35**)

To a solution of **46** (15 mg, 0.048 mmol) in DCM (0.5 mL) at 0 °C were added pyridine (4 µL, 0.05 mmol) and methanesulfonyl chloride (4 µL, 0.05 mmol). The mixture was warmed to room temperature for 1 h, then diluted with DCM, washed with 1 M HCl, dried and concentrated in vacuo. The crude product was purified by flash column chromatography (10–60% EtOAc in pentane) to produce **35** (10 mg, 0.025 mmol, 53%) as a white solid. ^1^H NMR (500 MHz, CDCl_3_) δ 7.81 (dd, *J* = 7.6, 2.3 Hz, 1H), 7.47 (dd, *J* = 7.8, 1.7 Hz, 1H), 7.45–7.35 (m, 3H), 7.26 (s, 2H), 6.66–6.62 (m, 1H), 4.75 (p, *J* = 6.9 Hz, 1H), 4.66 (s, 2H), 3.94 (s, 2H), 3.08 (s, 3H), 1.43 (d, *J* = 6.9 Hz, 6H); ^13^C NMR (125 MHz, CDCl_3_) δ 167.9, 153.9 (d, *J* = 246 Hz), 142.5, 141.1, 137.4 (d, *J* = 4 Hz), 131.0, 129.7, 125.7, 125.2, 125.13 (d, *J* = 8 Hz), 125.12, 122.0 (d, *J* = 61 Hz), 116.4 (d, *J* = 20 Hz), 68.1, 67.3, 50.7, 40.0, 21.5 (2 × C); HRMS (ESI^+^) C_19_H_22_FN_2_O_4_S^+^ ([M+H]^+^) requires 393.1279; found 393.1276.

**Scheme 7:**
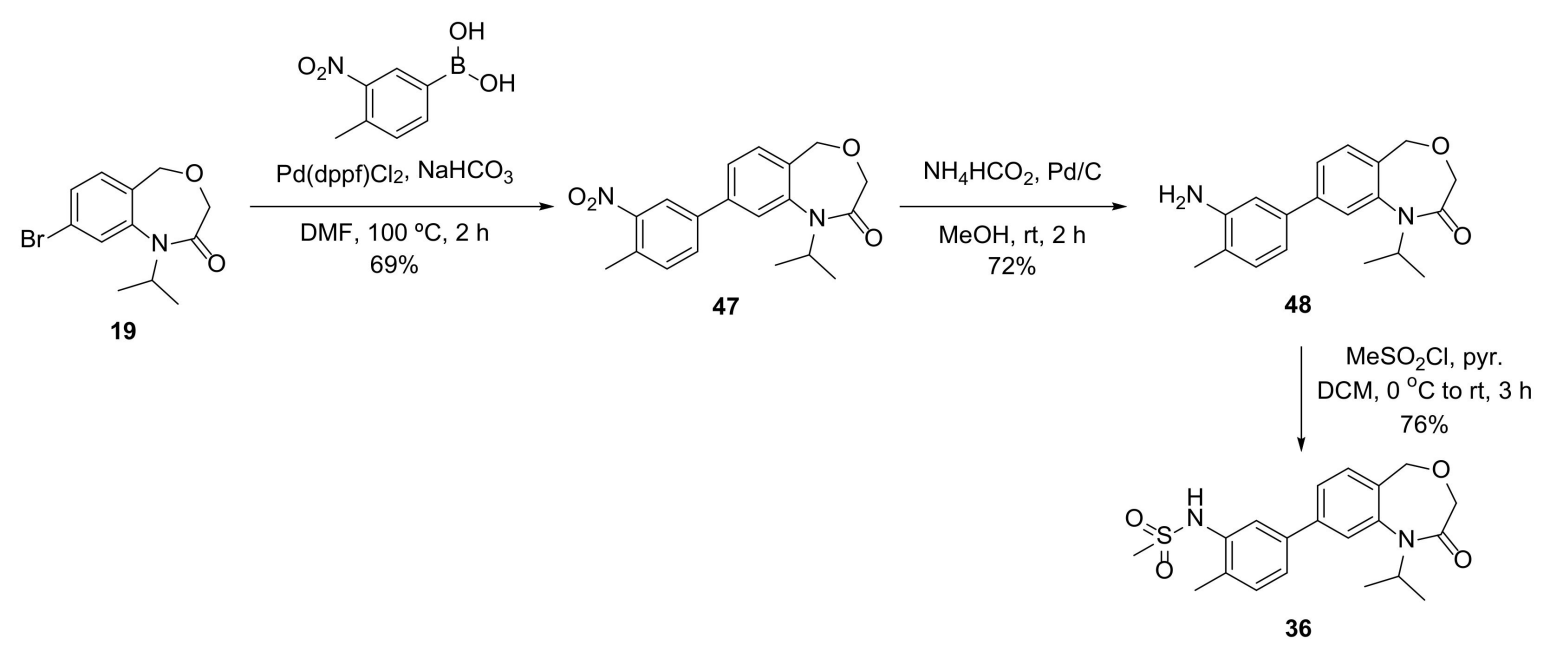
Synthesis of analogue **36**.

Compound **36** was prepared as shown in Scheme 7.

#### 4.14.39. 1-Isopropyl-8-(4-methyl-3-nitrophenyl)-1,5-dihydrobenzo[*e*][1,4]oxazepin-2(3*H*)-one (**47**)

General procedure D from **19** (58 mg, 0.20 mmol); yield 69% (48 mg, 0.14 mmol); white solid. ^1^H NMR (500 MHz, CDCl_3_) δ 8.19 (d, *J* = 2.0 Hz, 1H), 7.71 (dd, *J* = 8.0, 2.0 Hz, 1H), 7.52 (dd, *J* = 7.8, 1.7 Hz, 1H), 7.49–7.43 (m, 3H), 4.74 (hept, *J* = 6.9 Hz, 1H), 4.67 (s, 2H), 3.94 (s, 2H), 2.67 (s, 3H), 1.44 (d, *J* = 6.9 Hz, 6H); ^13^C NMR (125 MHz, CDCl_3_) δ 167.9, 149.7, 142.7, 140.3, 139.1, 133.6, 133.2, 131.3, 131.2, 130.1, 125.6, 123.1, 121.6, 68.1, 67.3, 50.9, 21.5, 20.2; HRMS (ESI^+^) C_19_H_21_N_2_O_4_^+^ ([M+H]^+^) requires 341.1496; found 341.1495.

#### 4.14.40. 8-(3-Amino-4-methylphenyl)-1-isopropyl-1,5-dihydrobenzo[*e*][1,4]oxazepin-2(3*H*)-one (**48**)

To a solution of **47** (40 mg, 0.12 mmol) and ammonium formate (8 mg, 0.12 mmol) in MeOH (3 mL) was added palladium on carbon (10% *w*/*w*, 4 mg). The flask was purged with hydrogen and the mixture stirred at room temperature for 2 h, then concentrated *in vacuo*. The crude product was purified by flash column chromatography (10–60% EtOAc in pentane) to produce **48** (27 mg, 0.087 mmol, 72%) as a white solid. ^1^H NMR (500 MHz, CDCl_3_) δ 7.47 (dd, *J* = 7.8, 1.7 Hz, 1H), 7.43 (d, *J* = 1.7 Hz, 1H), 7.37 (d, *J* = 7.8 Hz, 1H), 7.15 (d, *J* = 7.6 Hz, 1H), 6.92 (dd, *J* = 7.6, 1.9 Hz, 1H), 6.88 (d, *J* = 1.9 Hz, 1H), 4.74 (hept, *J* = 6.9 Hz, 1H), 4.65 (s, 2H), 3.94 (s, 2H), 3.74 (s, 2H), 2.23 (s, 3H), 1.42 (d, *J* = 6.9 Hz, 6H); ^13^C NMR (125 MHz, CDCl_3_) δ 168.0, 145.1, 143.2, 142.1, 138.9, 131.1, 130.6, 128.8, 125.6, 122.4, 121.6, 117.4, 113.4, 68.1, 67.4, 50.6, 21.5 (2 × C), 17.1; HRMS (ESI^+^) C_19_H_23_N_2_O_2_^+^ ([M+H]^+^) requires 311.1754; found 311.1753.

#### 4.14.41. *N*-(5-(1-Isopropyl-2-oxo-1,2,3,5-tetrahydrobenzo[*e*][1,4]oxazepin-8-yl)-2-methylphenyl)methanesulfonamide (**36**)

To a solution of **48** (19 mg, 0.061 mmol) in DCM (0.5 mL) at 0 °C were added pyridine (5 µL, 0.06 mmol) and methanesulfonyl chloride (5 µL, 0.06 mmol). The mixture was warmed to room temperature for 3 h, then diluted with DCM, washed with 1 M HCl, dried and concentrated *in vacuo*. The crude product was purified by flash column chromatography (10–60% EtOAc in pentane) to yield **36** (18 mg, 0.046 mmol, 76%) as a white solid. ^1^H NMR (500 MHz, CDCl_3_) δ 7.73 (d, *J* = 1.7 Hz, 1H), 7.50 (dd, *J* = 7.8, 1.7 Hz, 1H), 7.45 (d, *J* = 1.7 Hz, 1H), 7.41 (d, *J* = 7.8 Hz, 1H), 7.39–7.31 (m, 2H), 7.26 (s, 1H), 6.52 (s, 1H), 4.75 (hept, *J* = 6.9 Hz, 1H), 4.66 (s, 2H), 3.94 (s, 2H), 3.06 (s, 3H), 2.39 (s, 3H), 1.42 (d, *J* = 6.9 Hz, 6H); ^13^C NMR (125 MHz, CDCl_3_) δ 168.0, 142.3, 141.8, 139.3, 135.4, 131.8, 130.8, 129.8, 129.4, 125.6, 124.5, 121.7, 121.5, 68.0, 67.3, 50.6, 40.1, 21.5 (2 × C), 17.7; HRMS (ESI^+^) C_20_H_25_N_2_O_4_S^+^ ([M+H]^+^) requires 389.1530; found 389.1523.

#### 4.14.42. *N*-(5-(1-Isopropyl-2-oxo-1,2,3,5-tetrahydrobenzo[*e*][1,4]oxazepin-8-yl)-2-methoxyphenyl)methanesulfonamide (**37**)

General procedure D from **19** (30 mg, 0.11 mmol); yield 75% (32 mg, 0.079 mmol); off white solid. ^1^H NMR (500 MHz, CDCl_3_) 7.80 (d, *J* = 2.3 Hz, 1H), 7.48 (dd, *J* = 1.8, 7.8 Hz, 1H), 7.42 (d, *J* = 1.7 Hz, 1H), 7.38 (d, *J* = 8.0 Hz, 1H), 7.37 (dd, *J* = 2.0, 8.5 Hz, 1H), 7.02 (d, *J* = 8.5 Hz, 1H), 6.86 (brs, 1H), 4.75 (h, *J* = 7.0 Hz, 1H), 4.65 (s, 2H), 3.95 (s, 3H), 3.94 (s, 2H), 3.00 (s, 3H), 1.43 (d, *J* = 6.9 Hz, 6H); ^13^C NMR (125 MHz, CDCl_3_) 168.1, 149.6, 142.5, 141.7, 133.5, 130.9, 129.2, 126.7, 125.6, 124.2, 121.6, 119.9, 111.3, 68.2, 67.5, 56.2, 50.7, 39.4, 21.6; *m/z* (ESI^+^) 405.1 ([M+H]^+^, 100%); HRMS (ESI^+^) C_20_H_25_N_2_O_5_S^+^ ([M+H]^+^) requires 405.14787; found 405.14784.

#### 4.14.43. *N*-(3-(1-Isopropyl-2-oxo-1,2,3,5-tetrahydrobenzo[*e*][1,4]oxazepin-8-yl)-5-methoxyphenyl)methanesulfonamide (**38**)

General procedure D from **19** (30 mg, 0.11 mmol); yield 84% (36 mg, 0.089 mmol); white solid. ^1^H NMR (500 MHz, CDCl_3_) 7.48 (dd, *J* = 1.8, 7.7 Hz, 1H), 7.42 (s, 1H), 7.41 (d, *J* = 6.5 Hz, 1H), 6.99 (t, *J* = 1.7 Hz, 1H), 6.92 (dd, *J* = 2.3, 1.5 Hz, 1H), 6.85 (t, *J* = 2.1 Hz, 1H), 6.65 (s, 1H), 4.74 (h, *J* = 6.9 Hz, 1H), 4.66 (s, 2H), 3.94 (s, 2H), 3.89 (s, 3H), 3.08 (s, 3H), 1.43 (d, *J* = 6.9 Hz, 6H); ^13^C NMR (125 MHz, CDCl_3_) 168.1, 161.3, 142.9, 142.5, 142.0, 138.7, 131.0, 130.0, 125.9, 121.9, 111.5, 110.2, 105.3, 68.2, 67.5, 55.8, 50.9, 39.7, 21.6; *m/z* (ESI^+^) 405.1 ([M+H]^+^, 100%); HRMS (ESI^+^) C_20_H_25_N_2_O_5_SNa^+^ ([M+Na]^+^) requires 427.12981; found 427.12980.

**Scheme 8:**
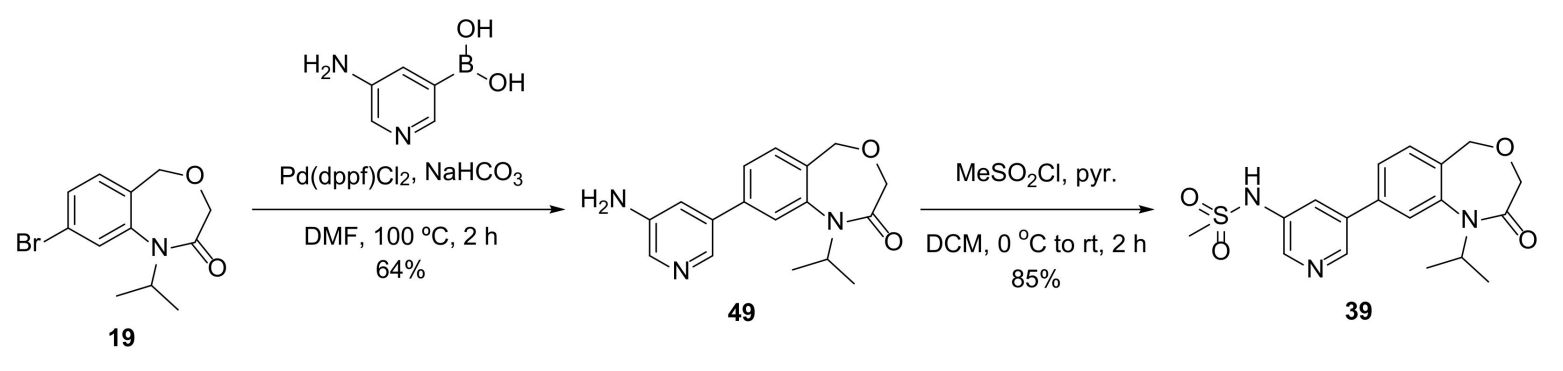
Synthesis of analogue **39**.

Compound **39** was prepared as shown in Scheme 8.

#### 4.14.44. 8-(5-Aminopyridin-3-yl)-1-isopropyl-1,5-dihydrobenzo[*e*][1,4]oxazepin-2(3*H*)-one (**49**)

General procedure D from **19** (30 mg, 0.11 mmol); yield 64% (20 mg, 0.07 mmol); yellow solid. ^1^H NMR (500 MHz, CDCl_3_) δ 8.25 (d, *J* = 1.9 Hz, 1H), 8.13 (d, *J* = 2.6 Hz, 1H), 7.47 (dd, *J* = 7.8, 1.7 Hz, 1H), 7.45–7.42 (m, 2H), 7.14 (t, *J* = 2.3 Hz, 1H), 4.74 (hept, *J* = 6.9 Hz, 1H), 4.66 (s, 2H), 3.94 (s, 2H), 3.84 (s, 2H), 1.43 (d, *J* = 6.9 Hz, 6H); ^13^C NMR (125 MHz, CDCl_3_) δ 167.9, 142.54, 142.51, 139.9, 138.5, 137.0, 135.8, 131.0, 129.9, 125.8, 121.7, 119.6, 68.1, 67.3, 50.8, 21.5 (2 × C); HRMS (ESI^+^) C_17_H_19_N_3_O_2_^+^ ([M+H]^+^) requires 298.1550; found 298.1549.

#### 4.14.45. *N*-(5-(1-Isopropyl-2-oxo-1,2,3,5-tetrahydrobenzo[*e*][1,4]oxazepin-8-yl)pyridin-3-yl)methanesulfonamide (**39**)

To a solution of **49** (15 mg, 0.050 mmol) in DCM (0.5 mL) at 0 °C was added pyridine (4 µL, 0.05 mmol) and methanesulfonyl chloride (4 µL, 0.05 mmol). The mixture was warmed to room temperature for 2 h, then diluted with DCM, washed with 1 M HCl, dried and concentrated *in vacuo*. The crude product was purified by flash column chromatography (10–60% EtOAc in pentane) to produce **39** (16 mg, 0.043 mmol, 85%) as a white solid. ^1^H NMR (500 MHz, CDCl_3_) δ 8.69 (s, 1H), 8.49 (s, 1H), 7.93 (t, *J* = 2.3 Hz, 1H), 7.55–7.44 (m, 3H), 7.07 (s, 1H), 4.76 (hept, *J* = 6.9 Hz, 1H), 4.68 (s, 2H), 3.95 (s, 2H), 3.12 (s, 3H), 1.44 (d, *J* = 6.9 Hz, 6H); ^13^C NMR (125 MHz, CDCl_3_) δ 167.9, 145.0, 142.8, 141.3, 138.4, 136.4, 133.9, 131.3, 130.6, 126.4, 125.9, 121.9, 68.1, 67.3, 50.8, 40.3, 21.5 (2 × C); HRMS (ESI^+^) C_18_H_22_N_3_O_4_S^+^ ([M+H]^+^) requires 376.1326; found 376.1325.

**Scheme 9:**
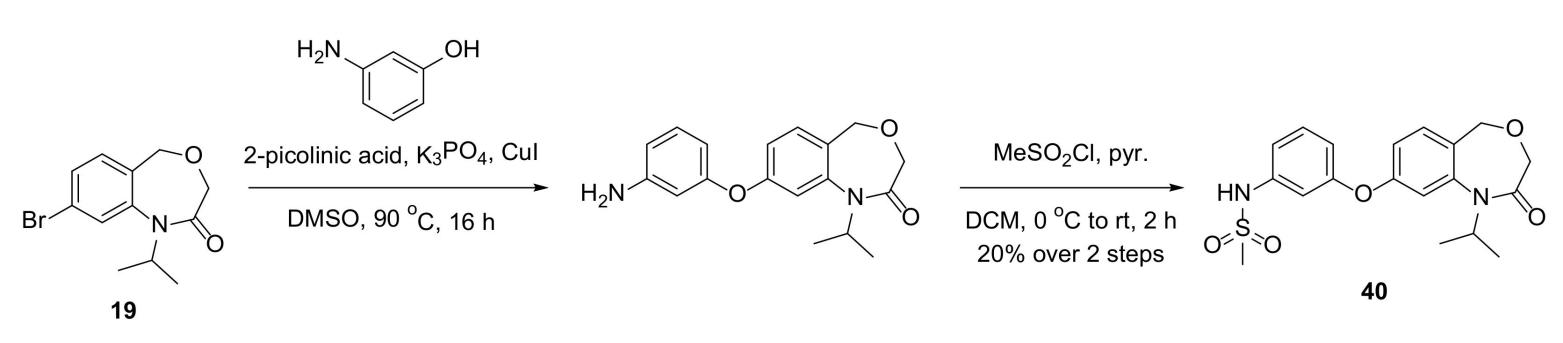
Synthesis of analogue **40**.

Compound **40** was prepared as shown in Scheme 9.

#### 4.14.46. *N*-(3-((1-Isopropyl-2-oxo-1,2,3,5-tetrahydrobenzo[*e*][1,4]oxazepin-8-yl)oxy)phenyl)methanesulfonamide (**40**)

To a flame-dried vial under argon were added **19** (47 mg, 0.17 mmol), 3-aminophenol (22 mg, 0.20 mmol), 2-picolinic acid (4 mg, 0.03 mmol), potassium phosphate (70 mg, 0.33 mmol) and copper iodide (3 mg, 0.02 mmol), followed by DMSO (2 mL). The mixture was heated to 90 °C for 16 h, then diluted with EtOAc, washed with water, NaHCO_3_, then brine, and the organic phase dried and concentrated *in vacuo*. The crude product was purified by flash column chromatography (20–100% EtOAc in pentane) to produce an inseparable mixture of products which was used without further purification.

To a solution of the resulting mixture (23 mg) in DCM (0.5 mL) at 0 °C was added pyridine (4 µL, 0.05 mmol) and methanesulfonyl chloride (4 µL, 0.05 mmol). The mixture was warmed to room temperature for 2 h, then diluted with DCM, washed with 1 M HCl, dried and concentrated in vacuo. The crude product was purified by flash column chromatography (20–100% EtOAc in pentane) to produce **40** (13 mg, 0.033 mmol, 20% over two steps) as a white solid.

^1^H NMR (500 MHz, CDCl_3_) δ 7.35 (t, *J* = 8.0 Hz, 1H), 7.31 (d, *J* = 8.3 Hz, 1H), 7.00–6.96 (m, 2H), 6.95 (d, *J* = 2.4 Hz, 1H), 6.91 (dd, *J* = 8.3, 2.4 Hz, 1H), 6.83 (ddd, *J* = 8.3, 2.3, 1.0 Hz, 1H), 6.77 (s, 1H), 4.68 (hept, *J* = 6.9 Hz, 1H), 4.60 (s, 2H), 3.92 (s, 2H), 3.05 (s, 3H), 1.35 (d, *J* = 6.9 Hz, 6H); ^13^C NMR (125 MHz, CDCl_3_) δ 167.9, 157.6, 157.5, 143.3, 138.5, 131.6, 131.0, 125.7, 116.8, 115.38, 115.39, 113.6, 111.0, 68.0, 67.1, 50.5, 39.7, 21.3 (2 × C); HRMS (ESI^+^) C_19_H_23_N_2_O_5_S^+^ ([M+H]^+^) requires 391.1322; found 391.1314.

**Scheme 10:**
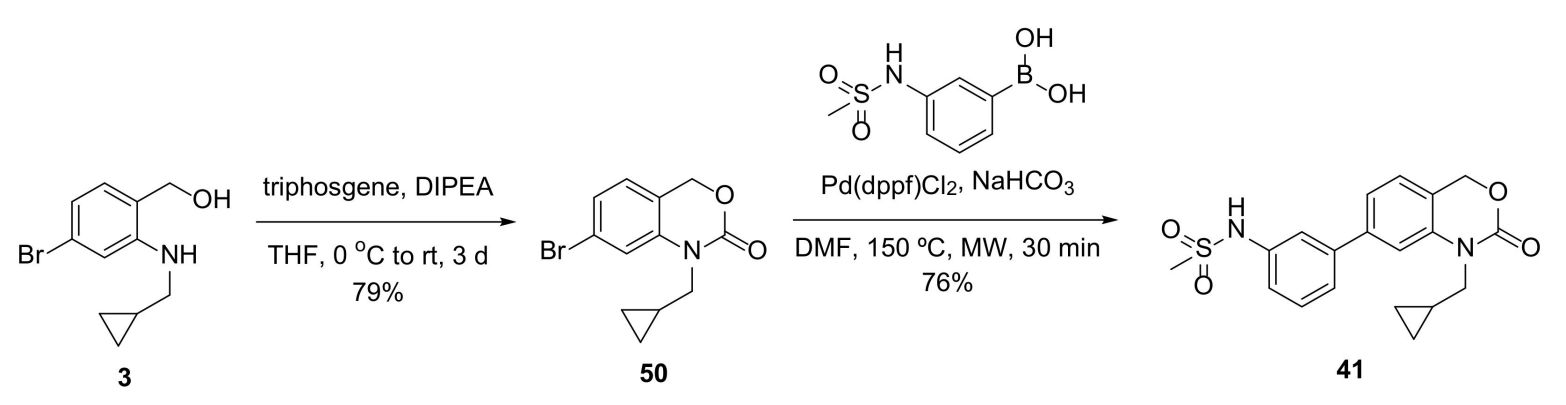
Synthesis of analogue **41**.

Compound **41** was prepared as shown in Scheme 10.

#### 4.14.47. 7-Bromo-1-(cyclopropylmethyl)-1,4-dihydro-2*H*-benzo[*d*][1,3]oxazin-2-one (**50**)

DIPEA (200 μL, 1.17 mmol) was added to a solution of **3** (100 mg, 0.390 mmol) and triphosgene (46 mg, 0.16 mmol) in THF (1.3 mL) at 0 °C. The mixture was stirred at room temperature for 3 days, then diluted with Et_2_O, filtered and diluted with EtOAc. The organic layer was washed with 1xNH_4_Cl and 1xbrine, dried over anhydrous Na_2_SO_4_ and concentrated *in vacuo*. The crude product was purified by flash column chromatography (5% to 15% EtOAc in pentane) to produce cyclised **50** (87 mg, 0.31 mmol, 79%) as a white solid. ^1^H NMR (400 MHz, CDCl_3_) δ 7.23–7.18 (m, 2H), 6.99 (d, *J* = 8.1 Hz, 1H), 5.15 (d, *J* = 0.8 Hz, 2H), 3.80 (d, *J* = 6.9 Hz, 2H), 1.23–1.12 (m, 1H), 0.62–0.53 (m, 2H), 0.48–0.41 (m, 2H); ^13^C NMR (100 MHz, CDCl_3_) 152.8, 139.1, 125.9, 125.8, 122.8, 119.7, 116.9, 67.1, 48.5, 9.5, 4.3; *m/z* (ESI^+^) 282.0 ([M+H]^+^, 100%); HRMS (ESI^+^) C_12_H_13_NO_2_Br^+^ ([M+H]^+^) requires 282.01242; found 282.01251.

#### 4.14.48. *N*-(3-(1-(Cyclopropylmethyl)-2-oxo-1,4-dihydro-2*H*-benzo[*d*][1,3]oxazin-7-yl)phenyl)methanesulfonamide (**41**)

General procedure D from **50** (13 mg, 0.046 mmol); yield 76% (13 mg, 0.035 mmol); white solid. ^1^H NMR (500 MHz, CDCl_3_) δ 7.46 (t, *J* = 7.8 Hz, 1H), 7.43 (t, *J* = 2.0 Hz, 1H), 7.39 (dt, *J* = 7.7, 1.4 Hz, 1H), 7.26 (s, 1H), 7.25–7.22 (m, 2H), 7.20 (d, *J* = 7.7 Hz, 1H), 6.61 (s, 1H), 5.26 (s, 2H), 3.91 (d, *J* = 6.8 Hz, 2H), 3.07 (s, 3H), 1.34–1.17 (m, 1H), 0.62–0.56 (m, 2H), 0.49 (dt, *J* = 6.4, 4.6 Hz, 2H); ^13^C NMR (125 MHz, CDCl_3_) δ 153.3, 142.5, 141.6, 138.3, 137.5, 130.5, 125.2, 124.3, 122.0, 120.5, 119.9, 119.5, 112.6, 67.3, 48.5, 39.8, 9.7, 4.5; *m/z* (ESI^+^) 373.1 ([M+H]^+^, 100%); HRMS (ESI^+^) C_19_H_21_N_2_O_4_S^+^ ([M+H]^+^) requires 373.12165; found 373.12177.

**Scheme 11:**
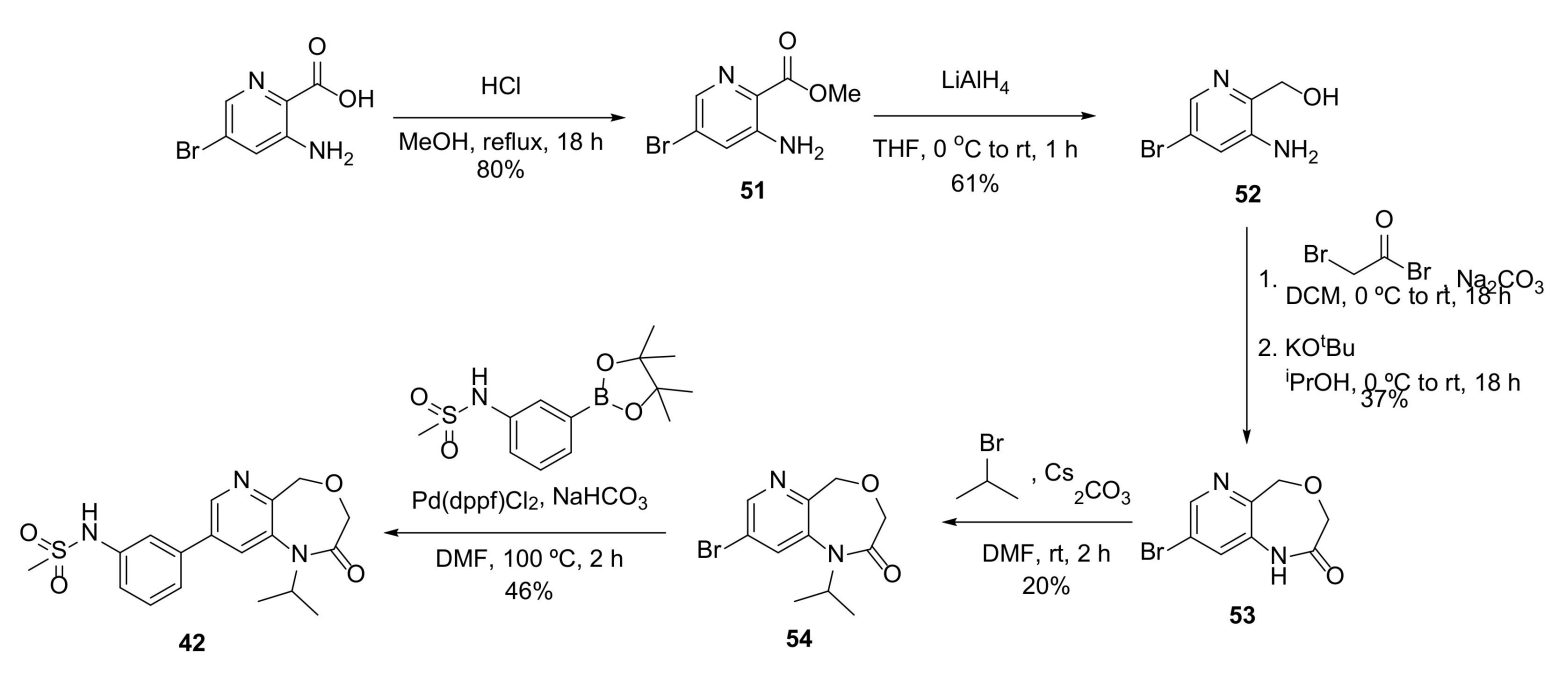
Synthesis of analogue **42**.

Compound **42** was prepared as shown in Scheme 11.

#### 4.14.49. Methyl 3-amino-5-bromopicolinate (**51**)

To a solution of 3-amino-5-bromo-pyridine-2-carboxylic acid (600 mg, 2.76 mmol) in MeOH (28 mL) was added 4 M HCl in 1,4-dioxane (2 mL). The reaction was refluxed for 18 h. Upon completion, the reaction was concentrated under vacuum and diluted with water. The aqueous layer was extracted with a mixture of CHCl_3_/IPA (3:1) and the combined organic layers were dried, filtered and concentrated under vacuum. The desired product **51** (512 mg, 2.22 mmol, 80%) was isolated as an off white solid and used in the following step without any further purification. ^1^H NMR (500 MHz, CDCl_3_) 8.06 (d, *J* = 2.0 Hz, 1H), 7.24 (d, *J* = 2.0 Hz, 1H), 5.82 (s, 2H), 3.97 (s, 3H); ^13^C NMR (125 MHz, CDCl_3_) 167.7, 147.6, 139.5, 126.6, 126.4, 125.4, 52.7; *m/z* (ESI^+^) 231.0 ([M+H]^+^, 100%); HRMS (ESI^+^) C_7_H_8_BrN_2_O_2_^+^ ([M+H]^+^) requires 230.97637; found 230.97668.

#### 4.14.50. (3-Amino-5-bromopyridin-2-yl)methanol (**52**)

General procedure B from **51** (250 mg, 1.08 mmol) with 1.2 equivalents of LiAlH_4_; yield 61% (135 mg, 0.660 mmol). The product was isolated with some minor impurities, but was taken to the following step and further purified thereafter. ^1^H NMR (400 MHz, CDCl_3_) δ 8.02 (d, *J* = 1.9 Hz, 1H), 7.13 (d, *J* = 1.9 Hz, 1H), 4.64 (d, *J* = 4.0 Hz, 2H), 3.91 (br s, 2H), 3.44 (t, *J* = 4.4 Hz, 1H); *m/z* (ESI^+^) 203.0 ([M+H]^+^, 25%).

#### 4.14.51. 8-Bromo-1,5-dihydropyrido[3,2-*e*][1,4]oxazepin-2(3*H*)-one (**53**)

General procedure C.2 from **52** (100 mg, 0.49 mmol); yield 37% (45 mg, 0.19 mmol); pale yellow solid. ^1^H NMR (500 MHz, CDCl_3_) 8.30 (d, *J* = 1.9 Hz, 1H), 8.02 (brs, 1H), 7.39 (d, *J* = 1.9 Hz, 1H), 4.90 (s, 2H), 4.59 (s, 2H); ^13^C NMR (125 MHz, CDCl_3_) 173.7, 146.4, 144.5, 133.2, 128.7, 119.5, 75.6, 74.0; *m/z* (ESI^+^) 243.0 ([M+H]^+^, 100%); HRMS (ESI^+^) C_8_H_8_BrN_2_O_2_^+^ ([M+H]^+^) requires 242.97637; found 242.97659.

#### 4.14.52. 8-Bromo-1-isopropyl-1,5-dihydropyrido[3,2-*e*][1,4]oxazepin-2(3*H*)-one (**54**)

To a suspension of **53** (42 mg, 0.17 mmol) in dry DMF (1.2 mL) were added Cs_2_CO_3_ (110 mg, 0.34 mmol) and 2-bromopropane (49 µL, 0.52 mmol). The mixture was stirred at room temperature for 2 h and then the reaction quenched with water. The aqueous layer was extracted with EtOAc and the combined organic layers were washed with brine, dried and concentrated under vacuum. The residue was purified by flash column chromatography (7–15% EtOAc in pentane) to produce the desired product **54** (10 mg, 0.035 mmol, 20%) as a pale-yellow solid. ^1^H NMR (500 MHz, CDCl_3_) 8.59 (d, *J* = 2.0 Hz, 1H), 7.74 (d, *J* = 2.0 Hz, 1H), 4.77 (s, 2H), 4.67 (hept, *J* = 6.9 Hz, 1H), 3.96 (s, 2H), 1.40 (d, *J* = 6.9 Hz, 6H); ^13^C NMR (125 MHz, CDCl_3_) 167.5, 148.9, 148.6, 139.0, 133.0, 120.6, 69.2, 68.5, 51.4, 21.4; *m/z* (ESI^+^) 285.0 ([M+H]^+^, 100%); HRMS (ESI^+^) C_11_H_14_BrN_2_O_2_^+^ ([M+H]^+^) requires 285.02332; found 285.02332.

#### 4.14.53. *N*-(3-(1-Isopropyl-2-oxo-1,2,3,5-tetrahydropyrido[3,2-*e*][1,4]oxazepin-8-yl)phenyl)methanesulfonamide (**42**)

General procedure D from **54** (10 mg, 0.04 mmol); yield 46% (6 mg, 0.02 mmol); white solid. ^1^H NMR (500 MHz, CDCl_3_) 8.73 (d, *J* = 2.1 Hz, 1H), 7.72 (d, *J* = 2.0 Hz, 1H) 7.52 (t, *J* = 7.9 Hz, 1H), 7.47 (t, *J* = 2.0 Hz, 1H), 7.41 (dt, *J* = 7.8, 1.3 Hz, 1H), 7.30 (ddd, *J* = 8.1, 2.3, 1.0 Hz, 1H), 4.86 (s, 2H), 4.77 (h, *J* = 6.9 Hz, 1H), 4.01 (s, 2H), 3.08 (s, 3H), 1.44 (dd, *J* = 6.9 Hz, 6H); ^13^C NMR (125 MHz, CDCl_3_) 167.8, 149.4, 146.3, 138.5, 138.2, 137.9, 136.8, 130.9, 128.9, 124.3, 120.6, 119.4, 69.5, 68.6, 50.9, 40.0, 21.7; *m/z* (ESI^+^) 376.1 ([M+H]^+^, 100%); HRMS (ESI^+^) C_18_H_22_N_3_O_4_S^+^ ([M+H]^+^) requires 376.13255; found 376.13254.

## Figures and Tables

**Table 1:** Structure-activity and structure-property relationships for analogues substituted at *N*-1.

Compound	R	EC_50_ [nM] ^a^	ER in mS9 ^b^	Solubility [µM] ^c^	clogP ^d^	LipE ^e^
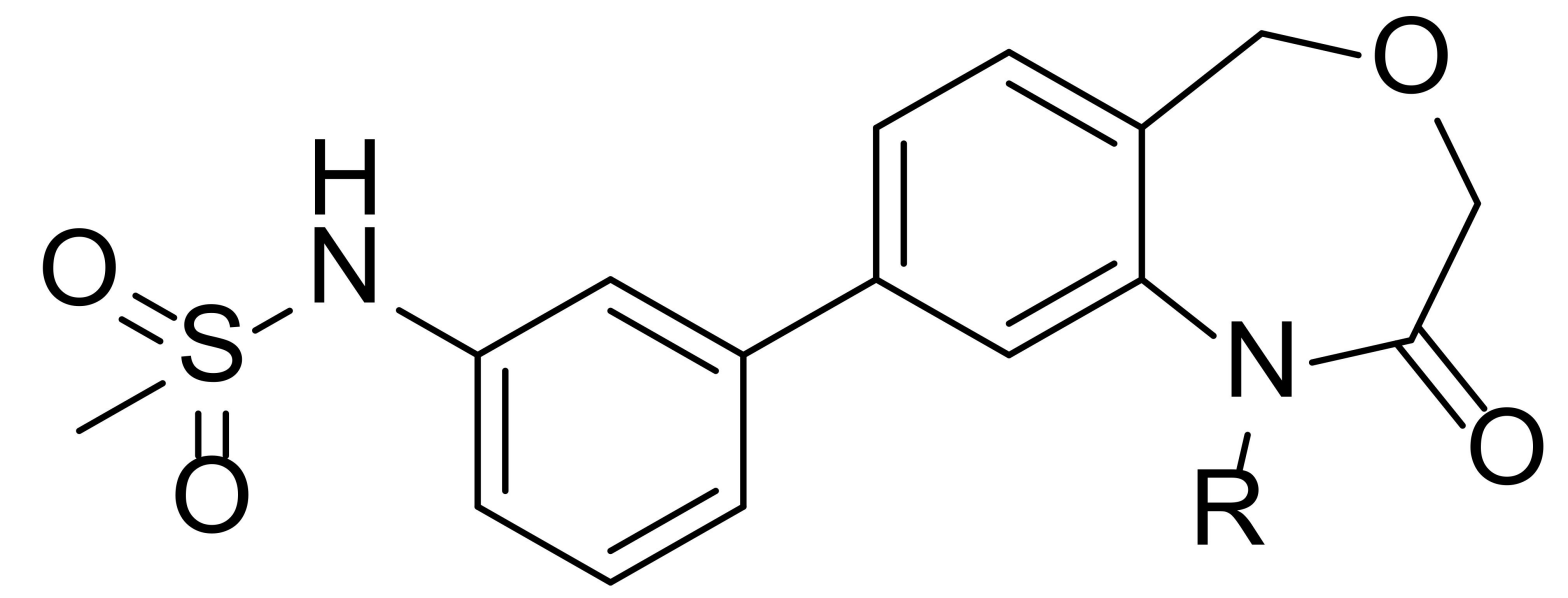						
OXS003976 (**5**)	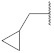	960	0.54	>200	1.7	4.3
**6**	-H	>10000	n.d. ^f^	>200	0.6	n.a. ^g^
**7**	-Me	>30000	0.07	>200	1.0	n.a.
**8**	-Et	6200	0.25	>200	1.4	3.8
OXS007002 (**9**)	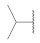	620	0.33	>200	1.7	4.5
**10**	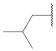	740	0.51	>200	2.1	4.0
**11**	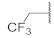	1900	0.05	33	1.9	3.8
**12**	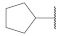	650	0.83	124	2.2	4.0
**13**	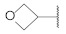	>10000	0.08	10	0.7	n.a.
**14**	-Ph	220	0.54	14	2.8	3.9

^a^ %CD11b response in HL60 cells; ^b^ extraction ratio (ER) = Cl_int_/species flow rate (mice: 90 mL/min/kg) in mouse S9 fraction (mS9); ^c^ semi-thermodynamic aqueous solubility; ^d^ calculated octanol-water partition coefficient clogP determined using Datawarrior; ^e^ lipophilic efficiency LipE = -LogEC_50_-cLogP; ^f^ not determined; ^g^ not applicable.

**Table 2:** Structure-activity and structure-property relationships for analogues substituted at the 8-position.

Compound	R	EC_50_ [nM] ^a^	ER in mS9 ^b^	Solubility [µM] ^c^	clogP ^d^	LipE ^e^
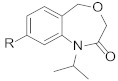						
**25**	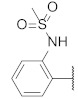	>30,000	n.d. ^f^	n.d.	1.7	n.a. ^g^
OXS007002 **9**	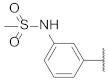	620	0.33	>200	1.7	4.5
**26**	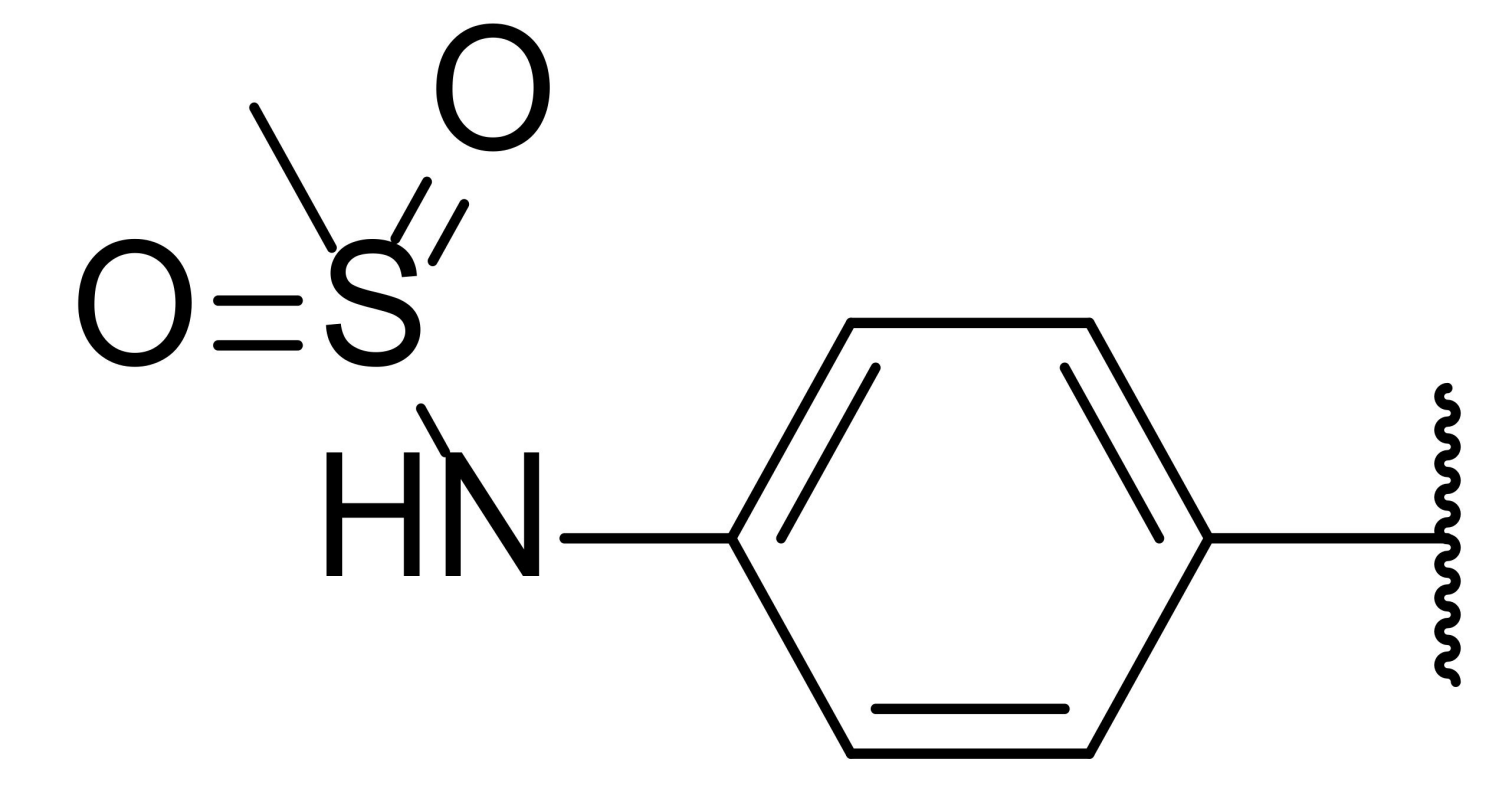	>30,000	n.d.	n.d.	1.7	n.a.
**27**	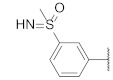	>30,000	n.d.	n.d.	2.2	n.a.
**28**	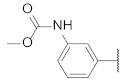	500	0.31	88	2.6	3.7
**29**	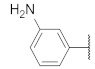	3300	n.d.	n.d.	2.0	3.5
**30**	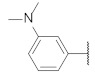	12,000	n.d.	n.d.	2.5	2.4
**31**	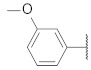	>10,000	n.d.	n.d.	2.6	n.a.
**32**	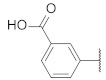	>30,000	n.d.	n.d.	2.2	n.a.
**33**	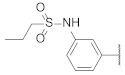	6800	n.d.	n.d.	2.7	2.5
**34**	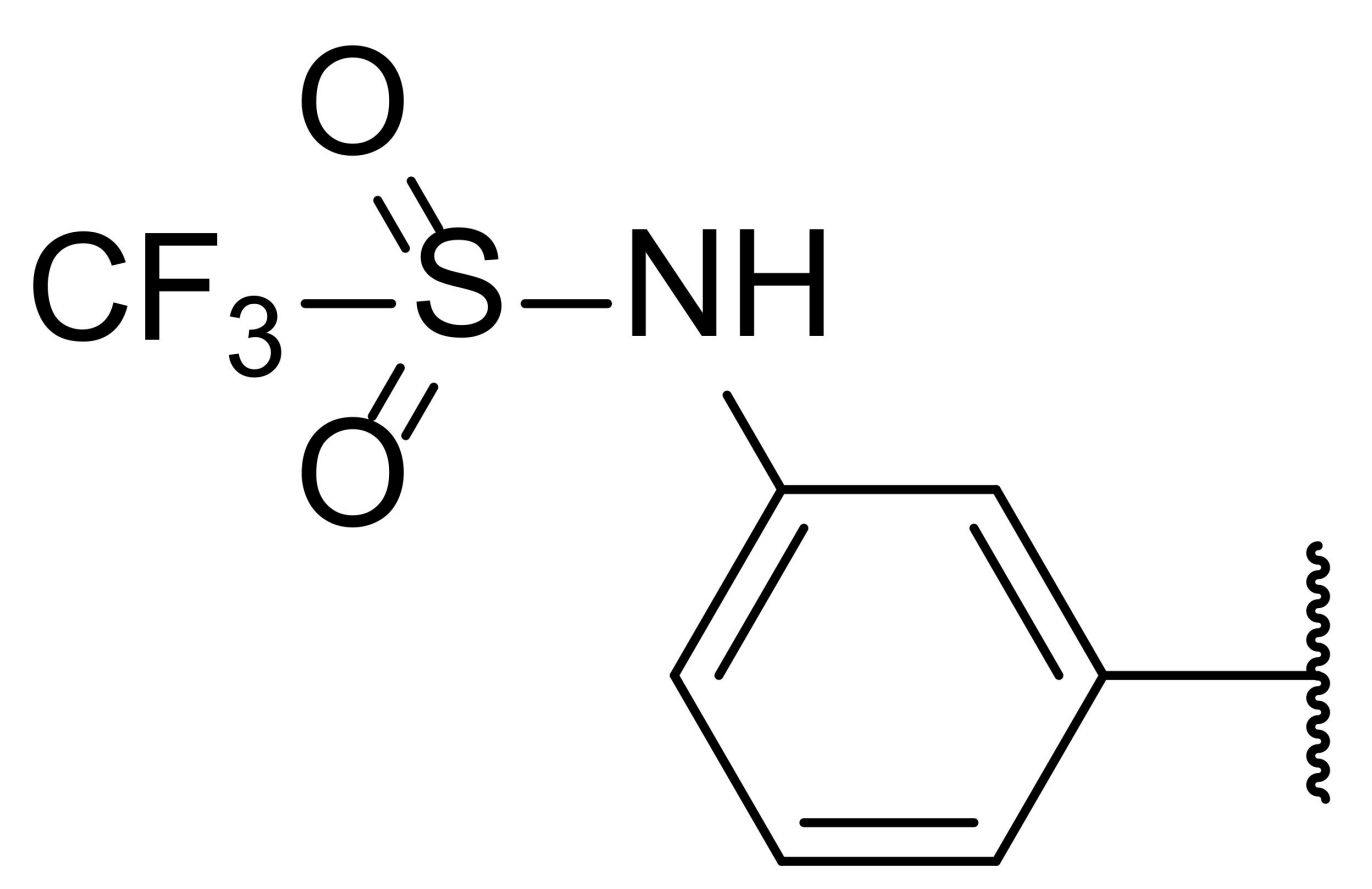	>10,000	n.d.	n.d.	3.8	n.a.
**35**	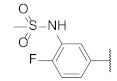	1600	0.21	>200	1.8	4.0
**36**	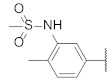	600	1	>200	2.1	4.1
**37**	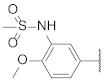	1200	1	>200	1.7	4.2
**38**	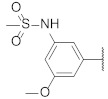	>30,000	n.d.	n.d.	1.7	n.a.
**39**	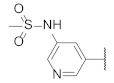	>30,000	n.d.	n.d.	0.7	n.a.
**40**	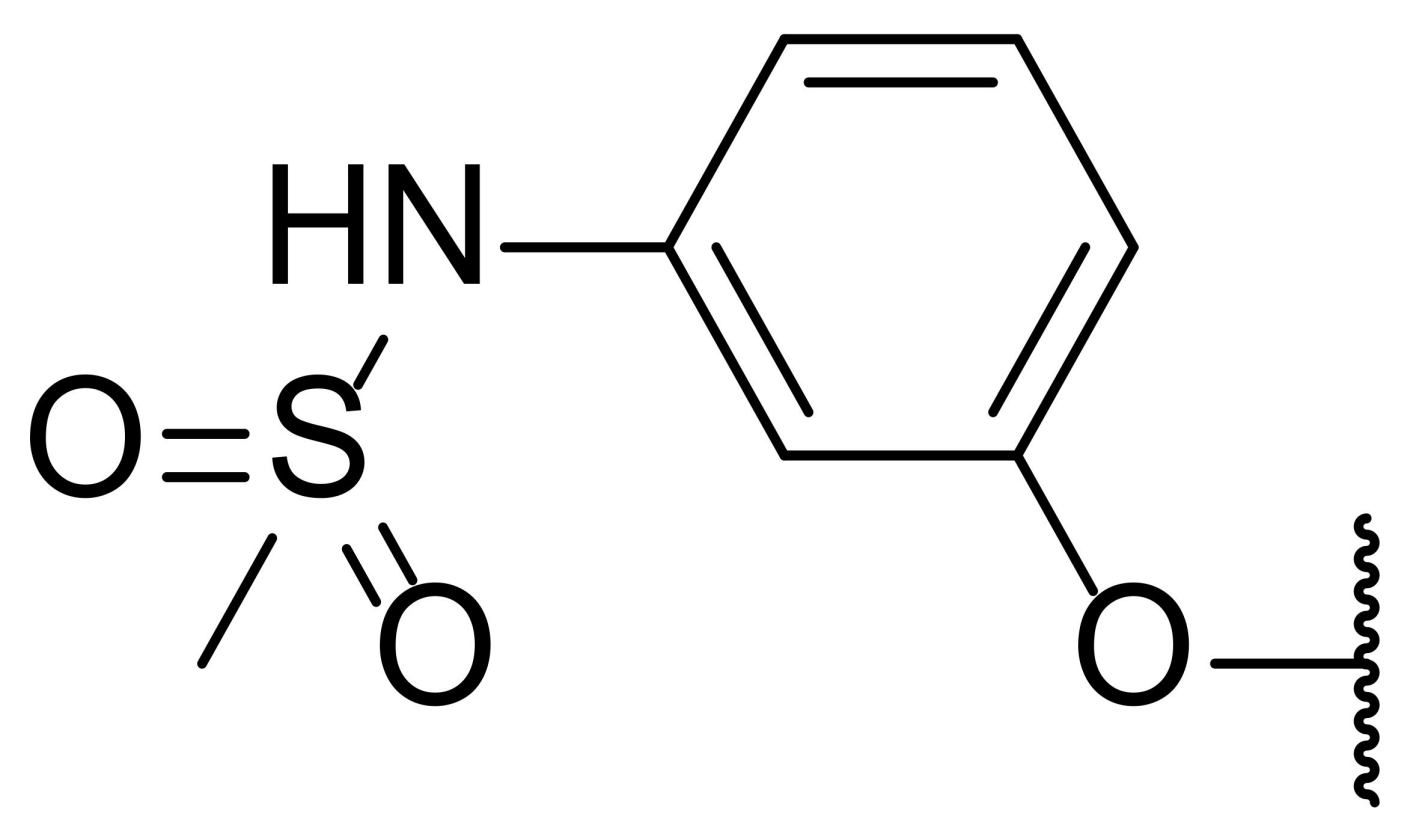	>30,000	n.d.	n.d.	1.5	n.a.

^a^ %CD11b response in HL60 cells; ^b^ extraction ratio (ER) = Cl_int_/species flow rate (mice: 90 mL/min/kg) in mouse S9 fraction (mS9); ^c^ semi-thermodynamic aqueous solubility; ^d^ calculated octanol-water partition coefficient clogP determined using Datawarrior; ^e^ lipophilic efficiency LipE = -LogEC_50_-cLogP; ^f^ not determined; ^g^ not applicable.

**Table 3:** Analogues with different cores structures.

Compound	Core	R	EC_50_ [nM] ^a^	ER in mS9 ^b^	Solubility [µM] ^c^	clogP ^d^	LipE ^e^
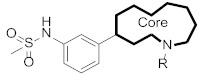							
**41**	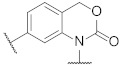	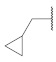	15,000	0.54	77	2.3	2.5
**42**	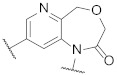	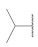	2200	0.09	>200	0.8	4.9

^a^ %CD11b response in HL60 cells; ^b^ extraction ratio (ER) = Cl_int_/species flow rate (mice: 90 mL/min/kg) in mouse S9 fraction (mS9); ^c^ semi-thermodynamic aqueous solubility; ^d^ calculated octanol-water partition coefficient clogP determined using Datawarrior; ^e^ lipophilic efficiency LipE = -LogEC_50_-cLogP.

## Data Availability

Not applicable.

## Supplementary Materials

The following are available online. Figure S1: Number of live cells per well and %viability of cell lines treated with either DMSO control or 10 μM OXS003976 over 4 days, determined with acridine orange and DAPI. Figure S2: Cytospins of OCI-AML3 cells treated with DMSO control or 10 µM OXS003976 stained with Modified Wright stain. Figure S3: Cytospins of THP-1 cells treated with DMSO control or 10 µM OXS003976 stained with Modified Wright stain. Figure S4: Cytospins of KG-1 cells treated with DMSO control or 10 µM OXS003976 stained with Modified Wright stain. Figure S5: Cytospins of Kasumi-1 cells treated with DMSO control or 10 µM OXS003976 stained with Modified Wright stain. Figure S6: Cytospins of ME-1 cells treated with DMSO control or 10 µM OXS003976 stained with Modified Wright stain Figure S7: Inhibition of tubulin polymerisation by (A) OXS007002 and (B) **17**.

## Sample Availability

Samples of the compounds are available from the authors.

## Abbreviations

AML: acute myeloid leukemia; APL, acute promyelocytic leukemia; ATRA, all-*trans* retinoic acid; clogP, calculated partition coefficient; C_max_, maximum concentration; Cl, clearance; DAPI, 4′,6-diamidino-2-phenylindole; DCM, dichloromethane; DHODH, dihydroorotate dehydrogenase; DMF, *N, N-*dimethylformamide; EfR, efflux ration; ER, extraction ratio; FLT, fms-like tyrosine kinase; IDH, isocitrate dehydrogenase; LipE, lipophilic efficiency; LSD, lysine-specific demethylase; mS9, mouse S9; MW, microwave irradiation; P_app_, apparent permeability; SAR, structure-activity relationship; SOC, standard of care; Sol, solubility; THF, tetrahydrofuran.
