# Supplementary material for: Identification and Preliminary Structure-Activity Relationship Studies of 1,5-Dihydrobenzo[e][1,4]oxazepin-2(3H)-ones That Induce Differentiation of Acute Myeloid Leukemia Cells In Vitro
Source: Molecules. 2021 Nov 2;26(21):6648. doi: 10.3390/molecules26216648 (PMC8588310; doi:10.3390/molecules26216648)
Supplement: Supplementary file 1 [file molecules-26-06648-s001.zip › molecules-1410129-supplementary.pdf]

SUPPLEMENTARY INFORMATION

Identification and Preliminary Structure-Activity Relationship Studies of 1,5-Dihydrobenzo[*e*][1,4]oxazepin-2(3*H*)-ones That Induce Differentiation of Acute Myeloid Leukemia Cells In Vitro

Laia Josa-Culleré [1,*], Thomas J. Cogswell [1], Irene Georgiou [1], Morgan Jay-Smith [1], Thomas R. Jackson [2], Carole J. R. Bataille [1,3], Stephen G. Davies [1], Paresh Vyas [2], Thomas A. Milne [2], Graham M. Wynne [1] and Angela J. Russell [1,3,*]

[1] Department of Chemistry, Chemistry Research Laboratory, University of Oxford, Mansfield Road, Oxford OX1 3TA, UK; tom.cogs1@gmail.com (T.J.C.); igeorgiou001@dundee.ac.uk (I.G.); morgan.jaysmith@outlook.com (M.J.-S.); carole.bataille@pharm.ox.ac.uk (C.J.R.B.); steve.davies@chem.ox.ac.uk (S.G.D.); graham.wynne@yahoo.co.uk (G.M.W.)

[2] MRC Molecular Haematology Unit, MRC Weatherall Institute of Molecular Medicine, NIHR Oxford Biomedical Research Centre Haematology Theme, Radcliffe Department of Medicine, University of Oxford, Oxford OX3 9DS, UK; thomas.jackson@paediatrics.ox.ac.uk (T.R.J.); paresh.vyas@imm.ox.ac.uk (P.V.); thomas.milne@imm.ox.ac.uk (T.A.M.)

[3] Department of Pharmacology, University of Oxford, Mansfield Road, Oxford OX1 3QT, UK

* Correspondence: laia.josacullere@gmail.com (L.J.-C.); angela.russell@chem.ox.ac.uk (A.J.R.)

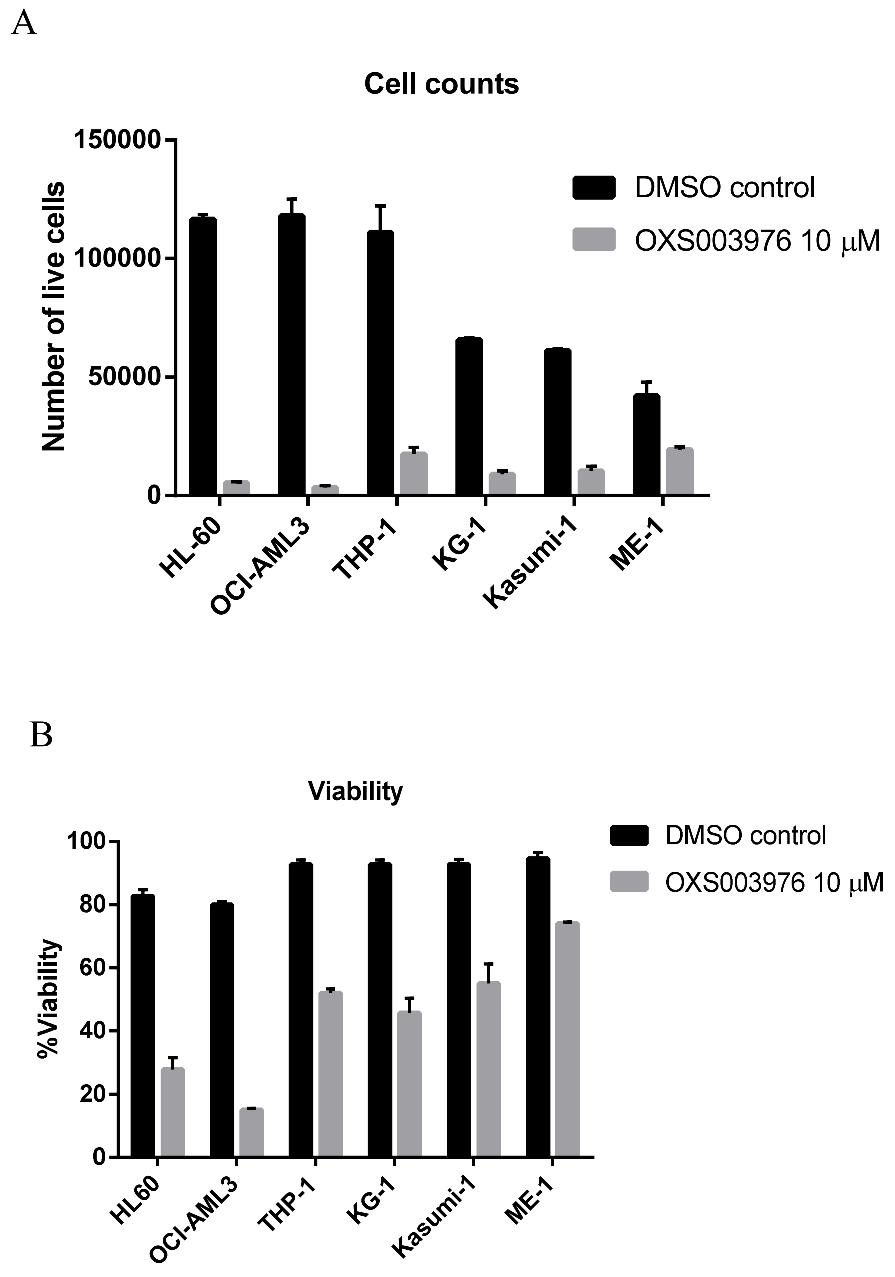

Figure S1. A) Number of live cells per well and B) %viability of cell lines treated with either DMSO control or 10 μM OXS003976 over 4 days, determined with acridine orange and DAPI.

A B

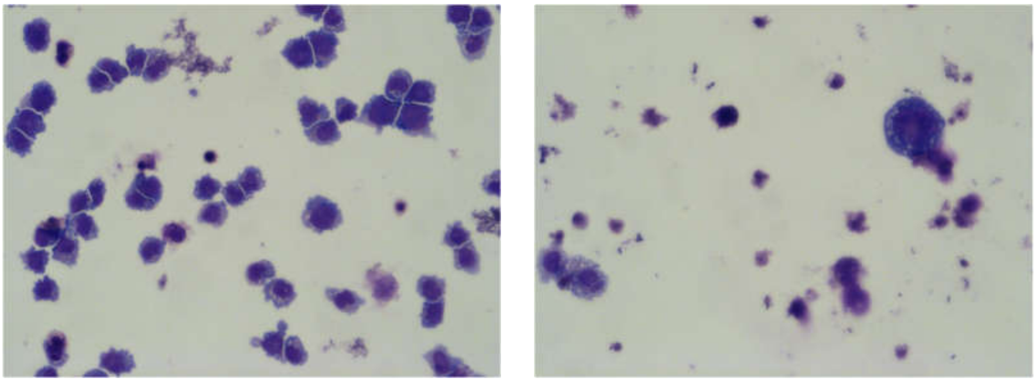

Figure S2. Cytospins of OCI-AML3 cells treated with (A) DMSO control or (B) 10 µM OXS003976 stained with Modified Wright stain.

A B

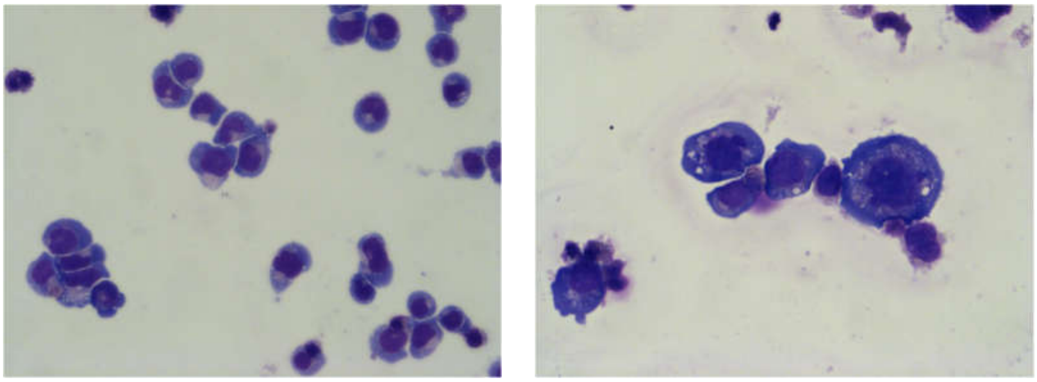

Figure S3. Cytospins of THP-1 cells treated with (A) DMSO control or (B) 10 µM OXS003976 stained with Modified Wright stain.

A B

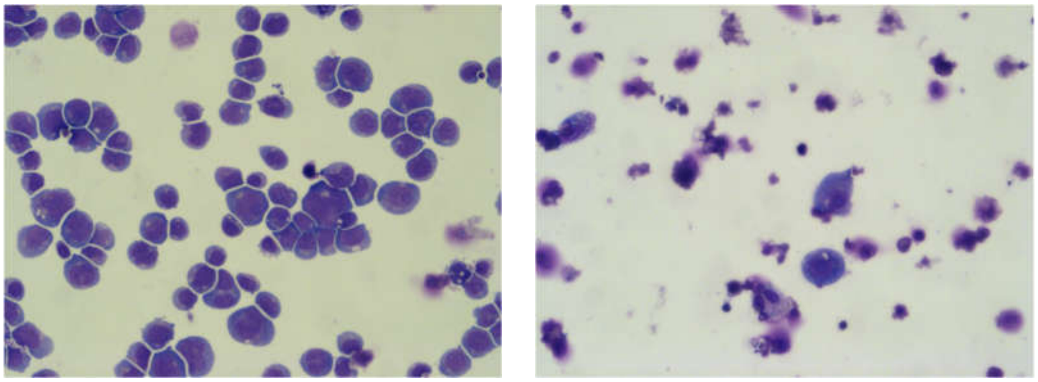

Figure S4. Cytospins of KG-1 cells treated with (A) DMSO control or (B) 10 µM OXS003976 stained with Modified Wright stain.

A B

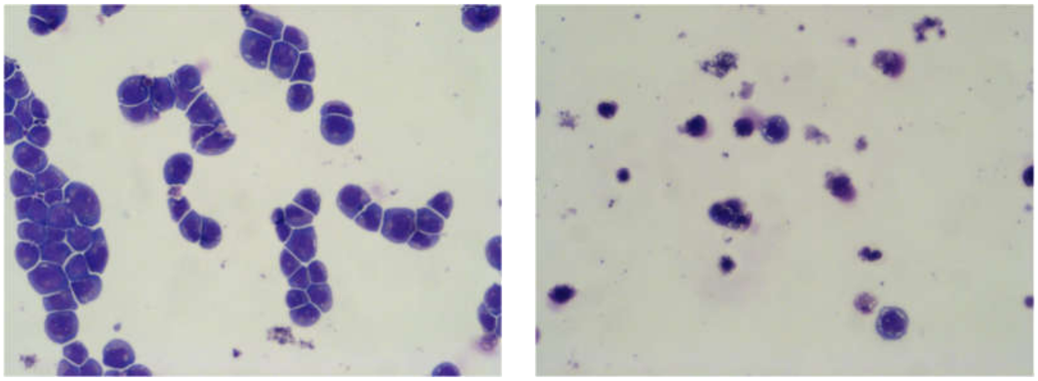

Figure S5. Cytospins of Kasumi-1 cells treated with (A) DMSO control or (B) 10 µM OXS003976 stained with Modified Wright stain.

A B

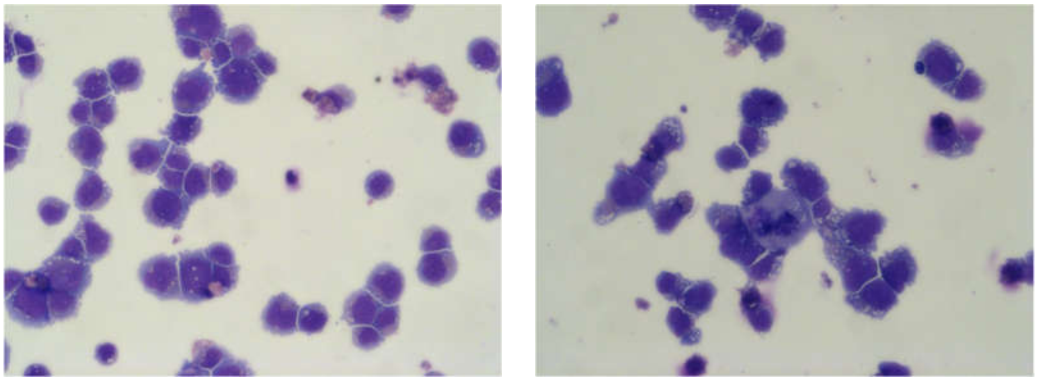

Figure S6. Cytospins of ME-1 cells treated with (A) DMSO control or (B) 10 µM OXS003976 stained with Modified Wright stain.

A B

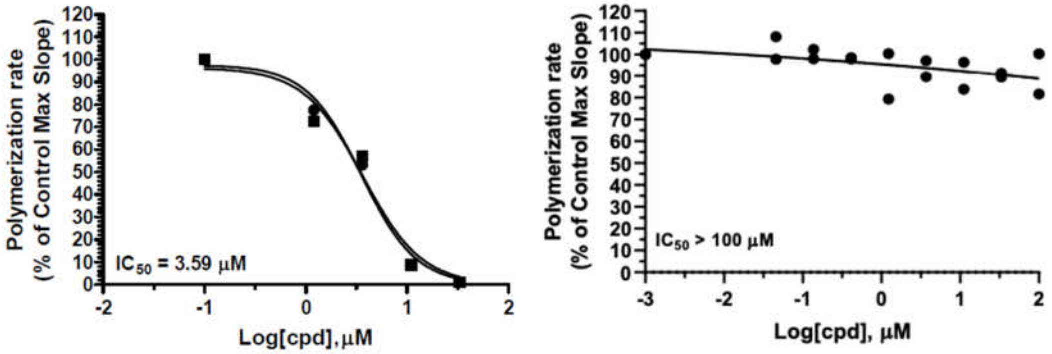

Figure S7. Inhibition of tubulin polymerisation by (A) OXS007002 and (B) **25**.
